# Spatiotemporal Prediction of Ideal Butterfly Habitats in Kun‐Ming's Urban Green Areas: Enabled by Maxent and ArcGIS

**DOI:** 10.1002/ece3.72300

**Published:** 2025-10-13

**Authors:** Xiaoli Zhang, Jiahai Zhao, Kaiyuan Yi, Di Yuan, Zhe Zhang

**Affiliations:** ^1^ College of Landscape Architecture and Horticulture Southwest Forestry University Kunming China

**Keywords:** butterfly habitat, ecological sensitivity, Maxent model, spatiotemporal prediction

## Abstract

This study presents groundbreaking insights into urban butterfly conservation by developing an integrated modelling framework to predict habitat suitability under climate change. The research addresses three critical gaps in current knowledge: (1) the lack of robust methodologies for assessing subtropical urban Lepidoptera habitats, (2) insufficient understanding of microclimate‐mediated edge effects in fragmented landscapes and (3) the absence of predictive tools for climate‐adaptive conservation planning. Our analytical approach combined Maxent species distribution modelling with high‐resolution GIS sensitivity analysis, incorporating 64 georeferenced occurrence records and 22 environmental variables. The optimised model achieved exceptional predictive accuracy (AUC = 0.966), identifying temperature seasonality (Bio7, 30.8% contribution) and dry‐season precipitation (Bio17, 21.5%) as dominant habitat filters. Spatial projections revealed a previously undocumented habitat paradox: while high suitability core areas may expand by 138.73 km^2^ under optimal scenarios, total suitable habitat could contract by 53.89 km^2^ due to climate‐driven edge effects. Three key innovations emerge from this work: First, we established a novel protocol for urban biodiversity assessment that integrates climatic, topographic and anthropogenic variables. Second, we demonstrated the critical role of microclimate buffering in maintaining habitat refugia, particularly in the southwestern Guandu and northwestern Chenggong districts. Third, we developed a decision‐support framework that identifies priority conservation zones based on habitat stability thresholds. These findings advance ecological theory by quantifying the impacts of urban heat islands on species distributions, providing actionable tools for city planners. The habitat stability maps and climate‐resilience indicators developed in this study are currently being implemented in Kunming's urban green space master plan, demonstrating the immediate practical relevance of this research.

## Graphical abstract

Using Maxent and ArcGIS with 22 SPSS–selected environmental parameters, this study analysed current butterfly distributions in Kunming's urban green spaces, predicted changes under three climate scenarios (1970–2000, 2030s, 2050s) and generated ecological sensitivity data. The Maxent model (AUC 0.966) identified key temperature—and—precipitation—related factors and showed highly suitable areas in certain Kunming districts, with ecological sensitivity analysis highlighting similar regions. Despite the expansion of highly suitable areas, overall suitable habitats declined. Four roadside green spaces were unsuitable. The research guides urban butterfly conservation in Kunming.

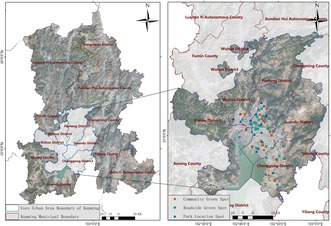

## 1 Introduction

Butterflies belonged to the two groups of *Glossata* and *Ritrysia*. Based on the characteristics of families, subfamilies and genera, the World Catalogue of Butterflies delineated the taxonomic status and distribution of butterflies. As an environmental indicator insect, butterflies were of great significance to forestry, agriculture and ecological conservation. Their sensitivity to environmental change made them an effective tool for monitoring ecosystem health and environmental change (Yan et al. 2006). The distribution, diversity and ecological characteristics of butterflies were closely related to environmental factors, and changes in their habitats can reflect changes in habitat quality (Song et al. 2025). The utilisation of butterflies as environmental indicator species has become a focal point in ecological research in recent years. For example, a study on butterfly diversity in urban green spaces in Beijing revealed that butterfly species diversity decreased with increasing urbanisation and was closely related to plant diversity and the size of the green space (Wang, Li, et al. 2023). Studies at the Xishuangbanna Tropical Botanical Garden had also found that the diversity of butterfly communities and their interactions with plants were highly sensitive to environmental changes (Wang et al. 2020). These results indicated that butterflies could not only reflect ecological changes but also provide an essential basis for biodiversity conservation and ecosystem management.

Recent assessments of global butterfly conservation status (IUCN Red List, Version 3.1, March 2024) revealed alarming trends across prominent taxonomic families. The *Papilionidae* exhibited particularly severe declines, with 4 species extinct, 22 critically endangered, 78 endangered and 86 vulnerable. Similar patterns emerged in other families: *Hesperiidae* (2 critically endangered), *Pieridae* (1 critically endangered), *Nymphalidae* (1 extinct, 5 critically endangered) and *Lycaenidae* (3 extinct, 14 critically endangered). These data underscored butterflies' precarious position as both biodiversity components and ecosystem service providers, particularly given their role in pollinating 65% of flowering plants. The crisis manifested most acutely in urban environments, where European grassland butterflies had declined by 53% since 1990, a trend mirrored in North America and Asia (Wang et al. 2020; Bartonova et al. 2017). Metropolitan areas exhibited 35%–40% species loss due to compounded effects of habitat fragmentation and microclimate alteration (Bartonova et al. 2020; Johansson et al. 2025). While the value of Lepidoptera as ecological indicators was well‐established, our understanding of the adaptive capacities of subtropical montane species remained critically limited. This knowledge gap, combined with the demonstrated inadequacy of traditional research methods, necessitated the development of innovative approaches to conservation science.

These pressing conservation challenges demanded innovative methodological approaches. Modern geospatial technologies, specifically the integrated ‘3S’ System (Remote Sensing/RS, Geographic Information System/GIS and Global Positioning System/GPS), offered transformative potential for butterfly conservation (Revathy and Sukanya 2025; Zhu et al. 2022). These technologies had been extensively applied in ecological research, environmental science and resource management, particularly in species distribution modelling, where they provided high‐precision environmental datasets and analytical tools (Li et al. 2022). Among various modelling approaches, ecological niche modelling had emerged as a critical methodology for assessing and predicting suitable habitats by synthesising species occurrence records with environmental variables (Wang and Chen 2004). Such models offered vital guidance for conservation planning through their capacity to analyse habitat requirements and project potential species distributions (Zhu and Qiao 2016; Zhu et al. 2013). Current niche modelling techniques encompassed several established methods: the BIOCLIM System for bioclimatic envelope analysis (Guisan and Zimmermann 2000), Random Forests (RFs) machine learning algorithms (Zimmermann et al. 2010), the Genetic Algorithm for Rule‐set Prediction (GARP) (Semwal et al. 2021) and the Maximum Entropy (MaxEnt) modelling framework (Ma, Ban, et al. 2021), each demonstrating distinct advantages across applications. Notably, the MaxEnt algorithm had been empirically validated for its superior performance in predicting climate‐change impacted species distributions (Haase et al. 2021; Hu et al. 2020), offering three key advantages: (1) reduced dependence on extensive occurrence records, (2) robust predictive accuracy and spatiotemporal scalability enabling projections across temporal and geographic dimensions (Tian et al. 2022; Wei and Xu 2020) and (3) enhanced precision through species‐specific parameter optimization (Ahmed et al. 2015; Vaz et al. 2015).

Although the Maxent model had been widely used to predict the distribution of suitable areas for many types of organisms, including *Yellow striped grey‐winged moth* (Muscarella et al. 2015), *Spodoptera litura* (Fang et al. 2022), *Steppe carabid* (Zhao et al. 2022), Birds (Liu, Bai, et al. 2022), *Red‐crowned Crane* Habitat (Ma, Wang, et al. 2021), *White Crane Wintering Ground* (H. T. Zhou 2016), *Chinese merganser* distribution area (Liu, Zhang, et al. 2024) and the Distribution of *Rhinoceros Leptospermatica* in China (Wang, Ding, et al. 2023), studying the suitable areas for butterflies and utilising the optimised Maxent model and ArcGIS for ecological sensitivity analysis could enhance the prediction accuracy and the scientific basis of environmental protection. Maxent predicted species distribution based on the principle of maximum entropy, and parameters affected accuracy. AUC (Area Under the Receiver Operating Characteristic Curve) was a key indicator for evaluating the predictive performance of species distribution models, with values ranging from 0.5 to 1.0. It quantified the model's ability to distinguish species presence from background points. ArcGIS analysed and visualised results, and with ecological sensitivity analysis, generated maps of suitable/sensitive areas. This combination predicted habitats, assessed changes, enabled multi‐species analysis and aided butterfly diversity research/protection strategies. Ecological sensitivity indicators reflected ecosystem vulnerability and conservation value; high sensitivity indicated greater environmental risks and species habitat uncertainty (Zhang et al. 2023), crucial for identifying optimal butterfly habitats. Research remained limited.

As sensitive bioindicators, butterflies played a pivotal role in monitoring ecosystem health and biodiversity conservation. Yunnan Province, a globally recognised biodiversity hotspot, harboured exceptional lepidopteran diversity; yet, systematic studies on urban butterfly assemblages remained scarce. Kunming's unique subtropical highland climate supported year‐round butterfly activity, making it an ideal location for studying these insects. Previous research by the Kunming Institute of Zoology had documented 2 new genera and 54 newly recorded species, revealing significant knowledge gaps in regional insect diversity. Our integrated approach combined field surveys across 64 habitat plots with Maxent modelling and GIS‐based ecological sensitivity analysis to: (1) quantify the dominant environmental factors (temperature and precipitation) governing butterfly distribution; (2) identify optimal habitats in Guandu's southwest, Chenggong's northwest and Xishan's northeast districts—areas requiring prioritised conservation; (3) analyse temporal changes showing expanding highly suitable areas but overall habitat decline by 2050s; and (4) optimise resource allocation by reducing inputs in low suitability areas (e.g., Luoyang Subdistrict). Field validation confirmed a 94% prediction accuracy, demonstrating the reliability of our model for conservation planning. This study established a scientific framework for optimising urban butterfly habitats, addressing critical challenges in biodiversity conservation within the context of climate change.

## 2 Materials and Methods

### 2.1 Study Area

Kunming was situated on the Yunnan‐Guizhou Plateau in Southwest China (102°10′–103°40′ E, 24°23′–26°22′ N), with a total area of 2100 km^2^ (urban core: 459 km^2^). The terrain generally sloped from higher elevations in the north (with an average altitude of approximately 2000 m) to lower elevations in the south. The downtown area was situated at around 1891 m. Characterised by a subtropical low‐latitude plateau monsoon climate, the region enjoyed an annual average temperature of approximately 15 degrees Celsius, with mild winters, cool summers, abundant sunshine and spring‐like conditions throughout the year, creating an ideal habitat for butterflies. Significant butterfly activity could still be observed even during autumn and winter (Liang et al. 2025; Ren et al. 2025). Recent surveys showed that in the five central urban districts of Kunming, namely Wuhua District, Panlong District, Xishan District, Guandu District and Chenggong District, butterflies were widely distributed in parks, residential areas and roadside green spaces, exhibiting high species diversity, richness and evenness, with well‐maintained ecosystem stability (Ouyang et al. 2000).

### 2.2 Sample Data Sources and Processing

**FIGURE 1:**
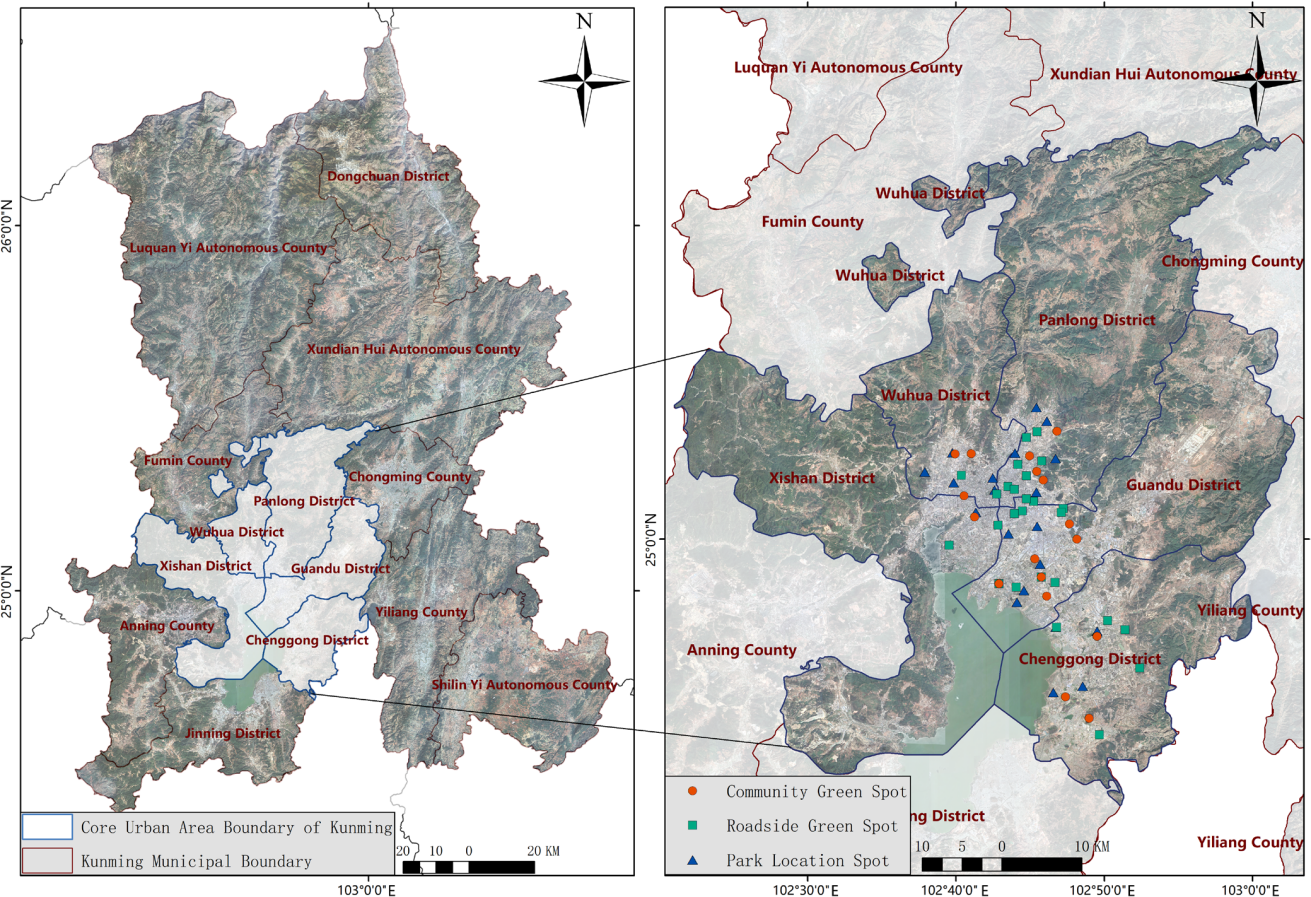
Study area location map. Plan approval number: Reproduction GS (2024) 0650—source osm website (https://www.openstreetmap.org/).

To enhance the research accuracy and reliability, we initially eliminated redundant distribution points and invalid data lacking precise geolocation information. The Maximum Entropy (Maxent) model exhibited several distinctive advantages: (1) minimal requirements for species occurrence data; (2) superior prediction accuracy; (3) excellent model scalability; and (4) temporal independence in sampling collection. Consequently, this modelling approach enabled the flexible application of prediction outcomes across diverse spatiotemporal scales, facilitating the practical evaluation of both current distributions and potential habitats for target species. Through systematic screening, we identified 64 representative butterfly occurrence points, comprising 20 park green spaces, 27 roadside greenbelts and 17 community green space observation sites (Table 1). Based on these standardised survey data, we successfully constructed the spatial distribution patterns of butterfly species (Figure 1). The geographical coordinates of each distribution point were precisely acquired using the Baidu Coordinate Picker. All species nomenclature and corresponding georeferenced data were systematically organised in Excel and exported in standardised CSV format (Hao et al. 2019).

**TABLE 1:** Butterfly distribution and quantity in Kunming urban green space in 2022.

Geographical position	Number of species
Community green space	
Next to the Yunnan Literature and Art Museum	15
Fivestar Garden	5
Olive home	18
Bozhong Garden	8
High‐tech neighbourhood	12
Garden of Milan	14
Southwest Forestry University	7
Wuhua Plaza	9
Dianchi International Convention and Exhibition Centre	24
Oriental Rose Garden	5
Yihui Garden	5
Heaven Yi peak view	14
Happy neighbourhood	9
Kunchuan Park	16
Grasse Perfume Town	12
Helen International	26
Yi LAN Court	46
Roadside green space	
Kunming Waterfall Park	7
Kunming Cuihu Park	33
Kunming Country Parks	27
Black Dragon Pool Park	19
Golden Hall Park	11
Lao Yu River Wetland Park	27
Crescent Lake Park	13
Lorong Park	5
Dounan Wetland Park	48
Tanhua Temple Park	19
Guandu Forest Park	6
Ruyi Park	37
Lianhuachi Park	22
Biji Park	25
Xi Hua Yuan	14
Sumishan Park	5
Dayu Park	24
Xiliangtang Wetland Park	31
Haihong Wetland Park	14
Longjiang Park	40
Kunming Waterfall Park	14
Kunming Cuihu Park	13
Kunming Country Parks	40
Black Dragon Pool Park	15
Golden Hall Park	12
Lao Yu River Wetland Park	9
Crescent Lake Park	8
Park green space	
Kunming Waterfall Park	127
Kunming Cuihu Park	72
Kunming Country Parks	632
Black Dragon Pool Park	131
Golden Hall Park	107
Lao Yu River Wetland Park	305
Crescent Lake Park	48
Lorong Park	100
Dounan Wetland Park	631
Tanhua Temple Park	28
Guandu Forest Park	37
Ruyi Park	159
Lianhuachi Park	18
Biji Park	30
Xi Hua Yuan	46
Sumishan Park	52
Dayu Park	446
Xiliangtang Wetland Park	164
Haihong Wetland Park	399
Longjiang Park	32

**FIGURE 2:**
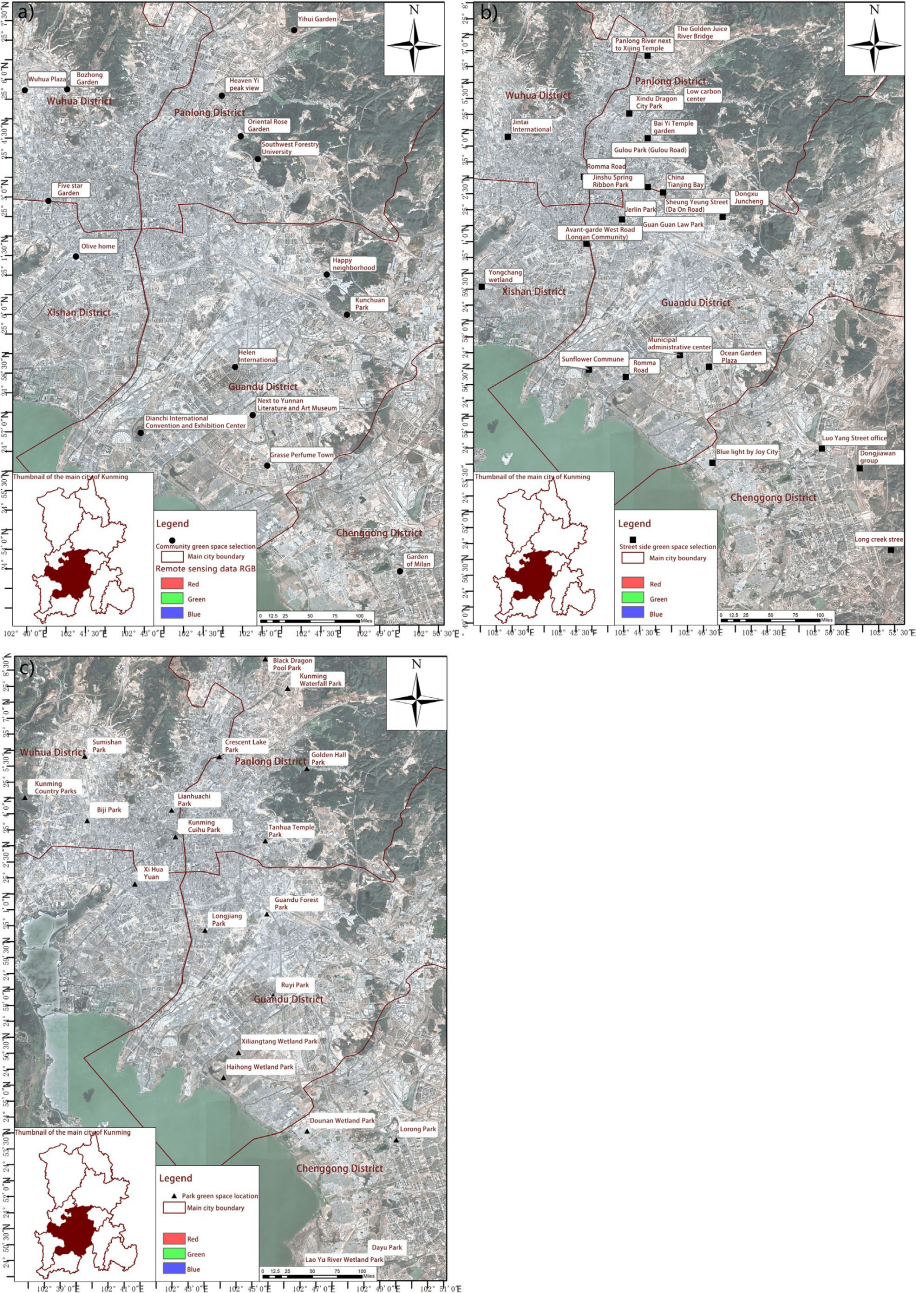
Spatial distribution of green space points. (a) Spatial distribution of community green space; (b) Spatial distribution of roadside green space; (c) Spatial distribution of park green space.

This study implemented standardised transect‐quadrat sampling across 64 sites in Kunming's urban core (20 park green spaces, 17 residential communities and 27 roadside habitats). Monthly surveys were conducted from January to December 2022 during optimal weather conditions (sunny/cloudy with wind speed < 3 m/s) at ≥ 20‐day intervals, recording all butterflies within standardised detection zones (2.5 m lateral × 5 m vertical × 5 m frontal) during peak activity periods (09:00–18:00). A total of 4347 specimens were collected (park: 3564 individuals representing 110 species/9 families; roadside: 538 individuals from 50 species/6 families; community: 245 individuals from 35 species/5 families) (Y. Zhou 2000), With taxonomic identification using authoritative references including *The Butterflies of China* (Sipei et al. 2025). Geospatial data included OGC‐compliant “Water by Note” map services (WeServer and Micro Map versions) and administrative boundary vectors from the Chinese Academy of Sciences' Resource and Environment Science and Data Center (www.resdc.cn). Utilising ArcGIS 10.8 software, we generated a comprehensive series of thematic maps illustrating the spatial distribution of butterflies in Kunming's urban core area, including: (1) park green space distribution maps; (2) roadside greenbelt distribution maps; (3) community green space distribution maps; and (4) integrated spatial distribution pattern maps (Figure 2).

### 2.3 Sources and Treatment of Environmental Factors

This study incorporated 19 bioclimatic variables (Bio1–Bio19) (Table 2) sourced from the WorldClim database (http://www.worldclim.org/) with a 20‐year temporal resolution. The BCC‐CSM2‐MR climate model was employed under three Shared Socioeconomic Pathways (SSP1‐2.6, SSP2‐4.5 and SSP5‐8.5) to analyse future climate scenarios. The selection of this model was based on its superior performance in simulating climatic conditions in Southwest China, particularly in terms of temperature and precipitation patterns under complex topography (Heinz and Prospero 2025; Li and Xia 2016). To address data gaps (2001–2020), ERA5 reanalysis data (0.25° × 0.25° resolution) from the European Centre for Medium‐Range Weather Forecasts (ECMWF) were integrated and validated against observational data from 12 meteorological stations in Kunming. All climate data were processed in ArcGIS 10.8, converted to ASCII format using SDMToolbox, and standardised to a 2.5 arc‐minute resolution.

**TABLE 2:** Information on bioclimatic variables.

Variable abbreviation	Description	Data source	Variable abbreviation	Description	Data source
Bio1	Annual mean temperature	WorldClim https://www.worldclim.org/	ALT	Elevation	Geospatial Data Cloud (http://www.gscloud.cn/)
Bio2	Mean diurnal range	LU	Aspect of slope	Based on elevation data extracted from ArcGIS
Bio3	Isothermy	RL	Stream density	
Bio4	Temperature seasonality	LU	Land use	Data Centre for Resources and Environmental Sciences, Chinese Academy of Sciences (https://www.resdc.cn/)
Bio5	The highest temperature in the hottest month	NPP	Net initial productivity	
Bio6	The lowest temperature in the coldest month	PD	Population density	ORNLLandscn (https://landscan.ornl.gov/citations)
Bio7	Annual temperature difference	NDVI	Vegetation normalised vegetation index	National Data Centre for Ecological Sciences (http://www.nesdc.org.cn/)
Bio8	The average temperature in the wettest quarter			
Bio9	The average temperature in the driest quarter			
Bio10	The average temperature in the hottest quarter			
Bio11	The average temperature in the coldest quarter			
Bio12	Annual precipitation			
Bio13	Precipitation in the wettest months			
Bio14	Precipitation in the driest months			
Bio15	Precipitation seasonality			
Bio16	Wettest season precipitation			
Bio17	Precipitation in the driest regions			
Bio18	Precipitation in the hottest season			
Bio19	Precipitation in the coldest season			

**FIGURE 3:**
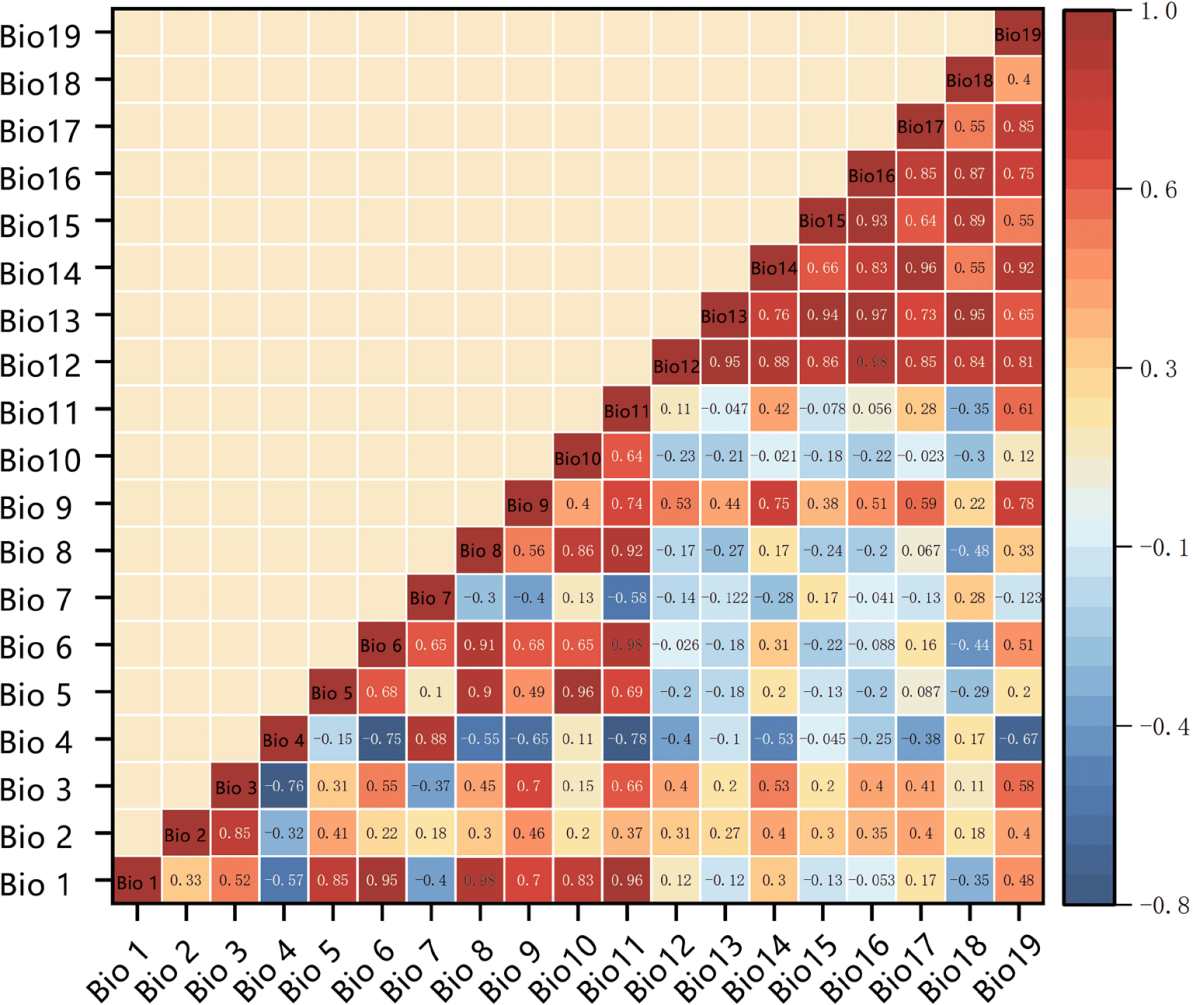
Heat map of the correlation of climate factors.

To optimise model performance and ensure ecological interpretability, we implemented a rigorous variable selection protocol by integrating seven supplementary environmental variables, altitude (ALT), slope (ASP), river density (RL), land use type (LU), net primary productivity (NPP), population density (PD) and normalised difference vegetation index (NDVI) alongside the 19 bioclimatic variables, all selected based on their documented relevance to butterfly habitat preferences (e.g., NDVI for host plant availability, altitude for thermal gradients). Prior to modelling, all 26 variables were standardised to a 2.5 arc‐minute resolution and evaluated for multicollinearity using Pearson correlation analysis (Figure 3), where variables exhibiting correlation coefficients ≥ |0.8| were flagged as highly collinear due to their potential to artificially inflate variable importance estimates and compromise model stability (Tian et al. 2022; Gan et al. 2022; Yu et al. 2022). To address this, we retained ecologically meaningful variables, excluded low‐contribution variables and ensured independence among predictors to avoid overfitting. This approach ultimately yielded a refined set of 22 variables (Table 3) that strike a balance between statistical robustness and ecological relevance by minimising redundancy while capturing key environmental gradients critical for species distribution modelling.

**TABLE 3:** Final biological variable information.

Variable abbreviation	Description	Variable abbreviation	Description
ALT	Elevation	Bio7	Annual temperature difference
RI	Stream density	Bio8	The average temperature in the wettest quarter
ASP	Aspect of slope	Bio9	The average temperature in the driest quarter
LU	Land use	Bio10	The average temperature in the hottest quarter
NDVI	Vegetation normalisation index	Bio11	The average temperature in the coldest quarter
PD	Population density	Bio12	Annual precipitation
NPP	Net initial productivity	Bio13	Precipitation in the wettest months
Bio2	Mean diurnal range	Bio14	Precipitation in the driest months
Bio3	Isothermy	Bio15	Precipitation seasonality
Bio4	Temperature seasonality	Bio17	Precipitation in the driest regions
Bio6	The lowest temperature in the coldest month	Bio19	Precipitation in the coldest season

### 2.4 MaxEnt **Modelling Approach**

The maximum entropy algorithm (MaxEnt) was rigorously implemented to assess the suitability of butterfly habitats under current and projected climate conditions. Georeferenced occurrence data (*n* = 64) underwent comprehensive quality control, including coordinate precision verification and removal of spatial duplicates using a 1‐km buffer, before being partitioned through stratified random sampling into training (75%, *n* = 48) and testing (25%, *n* = 16) subsets to maintain spatial representativeness (Ministry of Housing and Urban‐Rural Development of the People's Republic of China 2017; An et al. 2008). Model parameters were carefully optimised through systematic sensitivity analyses, with particular attention to feature classes and regularisation multipliers (Huang et al. 2023). A spatially explicit 10‐fold cross‐validation framework was employed, incorporating spatial blocking to account for autocorrelation effects. Each replicate ran 5000 iterations to ensure convergence. Model performance was evaluated using both threshold‐dependent (True Skill Statistic) and threshold‐independent (AUC) metrics, with final estimates derived from averaged results across all replicates to ensure robust inference (Molloy et al. 2014; Chai et al. 2025).

Habitat suitability predictions were categorised into four ecologically meaningful classes (Unsuitable: 0.0–0.1, Low: 0.1–0.3, Middle: 0.3–0.5, High: 0.5–1.0) based on the biological interpretability and statistical distribution of the output values (Mukherjee and Hossain 2024). Future projections were incorporated using statistically downscaled (1‐km resolution) climate data from the BCC‐CSM2‐MR model, representing three emission scenarios (SSP1‐2.6, SSP2‐4.5 and SSP5‐8.5) across 2021–2040 and 2041–2060 periods. Analytical procedures included: (1) spatial quantification of suitability class area changes; (2) centroid displacement analysis to track habitat shifts; (3) jackknife tests to evaluate variable importance; and (4) response curve analysis for key environmental drivers. This integrated analytical framework, which combines robust statistical validation with ecological interpretation, provides a comprehensive approach for assessing the impacts of climate change on species distributions while explicitly accounting for model uncertainty through spatially structured validation protocols (Anderson and Martinez‐Meyer 2004; Phillips et al. 2006).

### 2.5 Ecological Sensitivity

**FIGURE 4:**
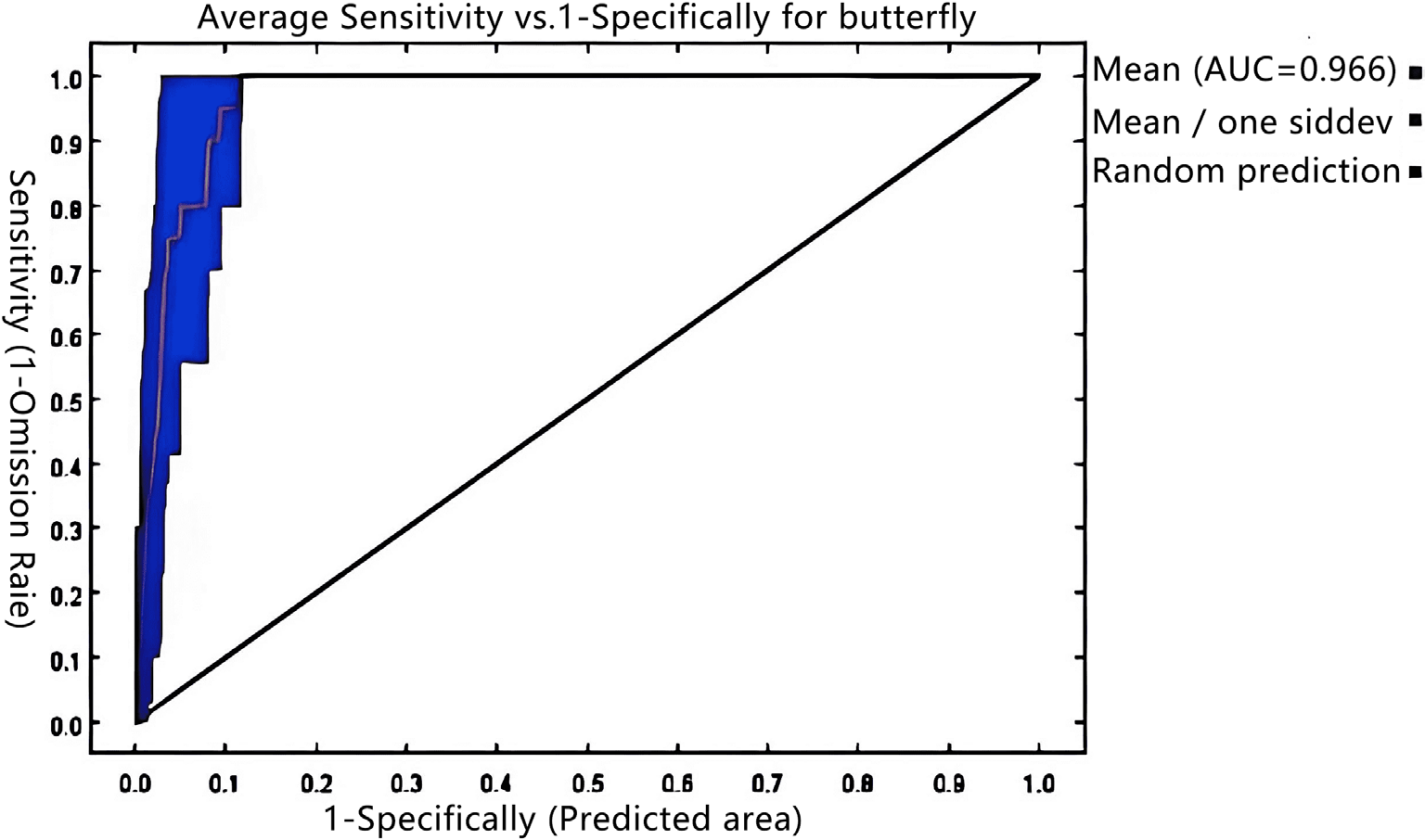
AUC value under the ROC curve.

Ecological sensitivity reflects an ecosystem's vulnerability to external disturbances and its conservation value, encompassing multiple dimensions, including climatic sensitivity, geological sensitivity, natural resource sensitivity and sensitivity to anthropogenic disturbances (Table 4). Using the Analytic Hierarchy Process (AHP) in ArcGIS (Table 5), we classified the study area into five sensitivity levels: insensitive, mildly sensitive, moderately sensitive, highly sensitive and extremely sensitive zones (Weng et al. 2019; Li et al. 2009). By integrating the Maxent model analysis of the 1970–2000 baseline period with projected data for 2021–2040 and 2041–2060, we were able to predict the spatial distribution patterns of suitable butterfly habitats and identify optimal survival zones. Through weighted overlay analysis of four sensitivity factors, we generated the spatial distribution characteristics of ecosystem sensitivity in Kunming's urban core (Figure 4). The ecological sensitivity index was calculated using the following weighted summation formula (Janitza et al. 2013):

$${\mathrm{EI}}_{i}=\sum_{j=1}^{n}\left(Y_{\mathrm{ij}}\times W_{j}\right)$$

where, ${\mathrm{EI}}_{i}$ is the ecological sensitivity index of the i evaluation unit; $Y_{\mathrm{ij}}$ is the standardised value of the j index of the i evaluation unit; $W_{j}$ is the weight of the j indicator, n indicates the total number of indicators. The value range of ${\mathrm{EI}}_{i}$ is [0,1]. The greater the ${\mathrm{EI}}_{i}$ value, the higher the degree of ecological sensitivity and the greater the probability of environmental problems in the region. The smaller the ${\mathrm{EI}}_{i}$ value, the lower the degree of ecological sensitivity, and the lower the probability of environmental problems in the region.

**TABLE 4:** Ecological sensitivity classification information.

Sort	Name	Source
Climate sensitivity	Mean air temperature	Data Centre for Resources and Environmental Sciences, Chinese Academy of Sciences (https://www.resdc.cn/)
	Relative humidity	
	Mean annual precipitation	National Oceanic and Atmospheric Administration (https://www.noaa.gov/)
Geological sensitivity	Elevation	Geospatial Data Cloud (http://www.gscloud.cn/)
	Slope	Based on elevation data extracted from ArcGIS
	Aspect of slope	
	Relief of relief	
	Topographic roughness	
	Surface cutting depth	
Natural resource sensitivity	Stream density buffer	Based on elevation data extracted from ArcGIS
	Lake extent	Geospatial Data Cloud (http://www.gscloud.cn/)
	Soil type	Data Centre for Resources and Environmental Sciences, Chinese Academy of Sciences (https://www.resdc.cn/)
	Land use type	
	Vegetation coverage	National Data Centre for Ecological Sciences (http://www.nesdc.org.cn/)
Human interference sensitivity	Road data	Data Centre for Resources and Environmental Sciences, Chinese Academy of Sciences (https://www.resdc.cn/)
	Population density	ORNL Landscn (https://landscan.ornl.gov/citations)

**TABLE 5:** Ecological sensitivity index factor weighting table.

Classification	Wi	Indicator Factors	Wi
Geological sensitivity	0.18	Elevation	0.34
		Slope	0.21
		Aspect	0.11
		Terrain relief	0.11
		Surface incision depth	0.15
		Topographic roughness index	0.08
Climate sensitivity	0.17	Mean temperature	0.17
		Annual mean precipitation	0.60
		Relative humidity	0.23
Natural resource sensitivity	0.31	River buffer zone	0.21
		Lake riparian buffer	0.18
		Soil taxonomy	0.11
		Land use	0.26
		Vegetation coverage	0.24
Sensitivity to human disturbance	0.34	Distance to roads	0.58
		Population density	0.42

## 3 Results and Analysis

### 3.1 Model Analysis

**FIGURE 5:**
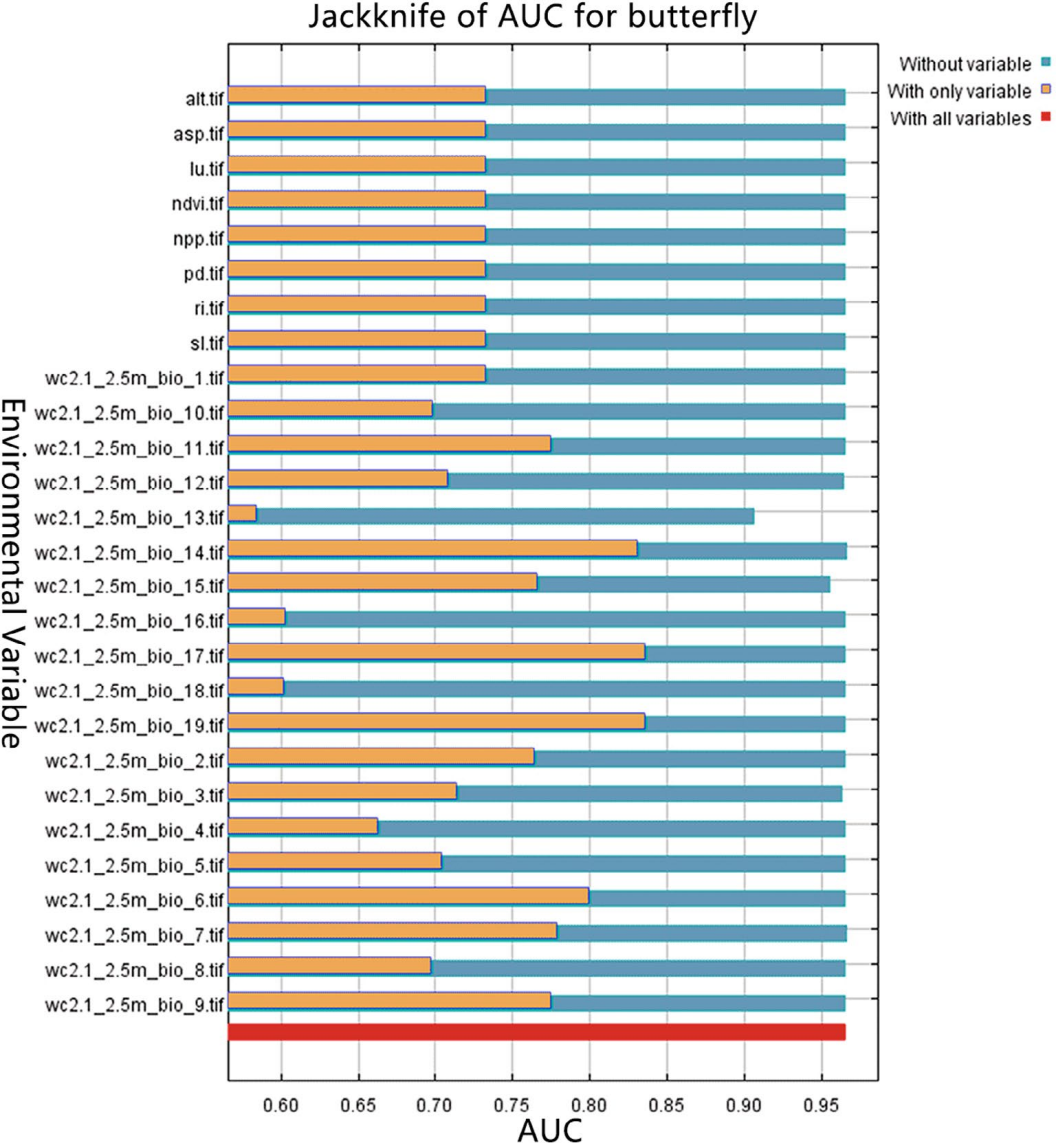
Cutting method to test the environment variable gain.

The Maxent model demonstrated excellent performance in predicting butterfly distribution across Kunming's urban green spaces (mean AUC = 0.966) (Figures 4 and 5). Based on 64 occurrence points and 22 environmental variables, the results identified six key climatic factors contributing 86.5% to the model (Tables 3 and 6). Among these, annual temperature range (Bio7) showed the highest contribution rate (30.8%), with annual temperature range (Bio7, 30.8%) being the most influential. This high contribution reflects butterflies' ectothermic sensitivity to thermal fluctuations, particularly under the combined effects of Kunming's monsoon climate and urban heat islands. Followed by precipitation of the driest quarter (Bio17, 21.5%), mean diurnal temperature range (Bio2, 12.6%), precipitation of the wettest month (Bio13, 12.5%), annual precipitation (Bio12, 5.2%) and precipitation of the coldest quarter (Bio19, 3.9%). The dominance of temperature and precipitation variables (collectively 86.5%) reflects butterflies' ectothermic sensitivity to thermal fluctuations and moisture‐dependent resource availability (Sun et al. 2012), particularly under Kunming's monsoon climate and urban heat island effects. In contrast, non‐climatic factors exhibited minimal influence. Topographic variables (elevation, slope and aspect) collectively contributed only 6.2%, likely due to Kunming's limited elevation range (1890–2000 m). Anthropogenic factors (land use type, population density and distance to roads) accounted for 4.8%, suggesting that butterflies are adaptable to urban disturbances. Productivity related variables (NDVI, NPP) contributed only 2.5%, possibly due to homogeneous vegetation management in urban green spaces. However, these secondary factors may interact with climate variables, for instance, specific slope aspects could enhance habitat suitability by altering local microclimates (Tian and Li 2024). These findings emphasise the importance of prioritising thermal‐moisture optimisation and microclimate heterogeneity in urban butterfly conservation.

**TABLE 6:** Contribution rate and displacement importance of significant environmental variables.

Environment variable	Contribution rate (%)	Permute important values (%)	Environment variable	Contribution rate (%)	Permute important values (%)
Bio7	30.8	0.4	Bio10	0.8	1.1
Bio17	21.5	0.9	Bio3	0.7	1.7
Bio2	12.6	2.6	LU	0.7	0
Bio13	12.5	42.7	NPP	0.7	0
Bio12	5.2	8.2	ASP	0.6	0
Bio19	3.9	0.4	PD	0.5	0
Bio15	2.7	29.2	RI	0.5	0
Bio4	1.9	0.6	Bio6	0.3	0
Bio14	1.7	0.2	Bio11	0.3	0
Bio8	0.9	12.1	ALT	0.3	0
Bio9	0.8	0	NDVI	0.2	0

**FIGURE 6:**
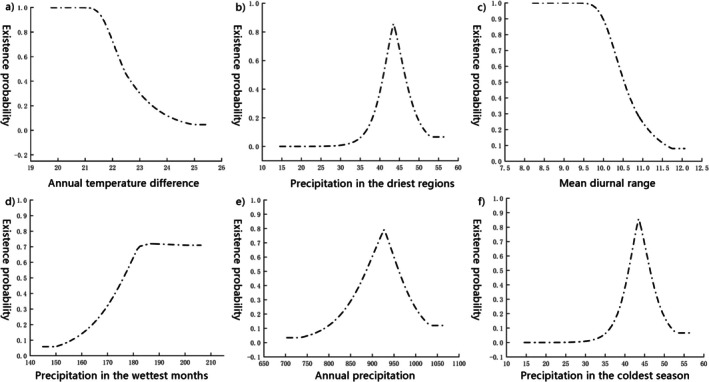
Average response curve of the significant environmental variable. (a) Annual temperature difference; (b) Precipitation in the driest regions; (c) Mean diurnal range; (d) Precipitation in the wettest months; (e) Annual precipitation; (f) Precipitation in the coldest season.

Impact of Environmental Variables on Butterfly Survival in Kunming. This study examined the impact of key environmental variables on the survival probability of butterflies across various green spaces in Kunming. Based on the habitat suitability threshold (0.239) derived from Alexander et al. (Zheng et al. 2016), optimal ranges for butterfly survival were identified using average response curves (Figure 6). The highest survival probability occurred within specific climatic intervals: annual temperature difference (Bio7, 19.5°C–21.5°C), precipitation in the driest regions (Bio17, 35–45 mm, peaking at 45 mm), mean diurnal range (Bio2, 8.0°C–9.55°C), precipitation in the wettest month (Bio13, 180–205 mm, peaking at 180 mm), annual precipitation (Bio12, 750–925 mm, peaking at 925 mm) and precipitation in the coldest quarter (Bio19, 35–43 mm, peaking at 43 mm).

The response curves reveal fine‐scale adaptations of butterflies to Kunming's climatic gradients. A moderate annual temperature difference (Bio7) likely optimises metabolic efficiency, facilitating essential activities such as foraging and reproduction. Precipitation in the driest regions (Bio17) peaks at 45 mm, suggesting that minimal moisture enhances resource availability without compromising arid adaptations. The optimal mean diurnal range (Bio2, 8.0°C–9.55°C) balances diurnal activity and nocturnal recovery, while peak precipitation in the wettest month (Bio13, 180 mm) sustains plant biomass without causing microhabitat saturation. High annual precipitation (Bio12, 925 mm) ensures ecosystem productivity, supporting the nutritional needs of both larvae and adults, whereas coldest‐quarter precipitation (Bio19, 43 mm) may mitigate the harshness of winter, benefiting overwintering stages. These thresholds collectively delineate the climatic niche maximising butterfly survival, providing critical insights for habitat conservation in Kunming's urban green spaces.

### 3.2 Sensitivity Analysis

**FIGURE 7:**
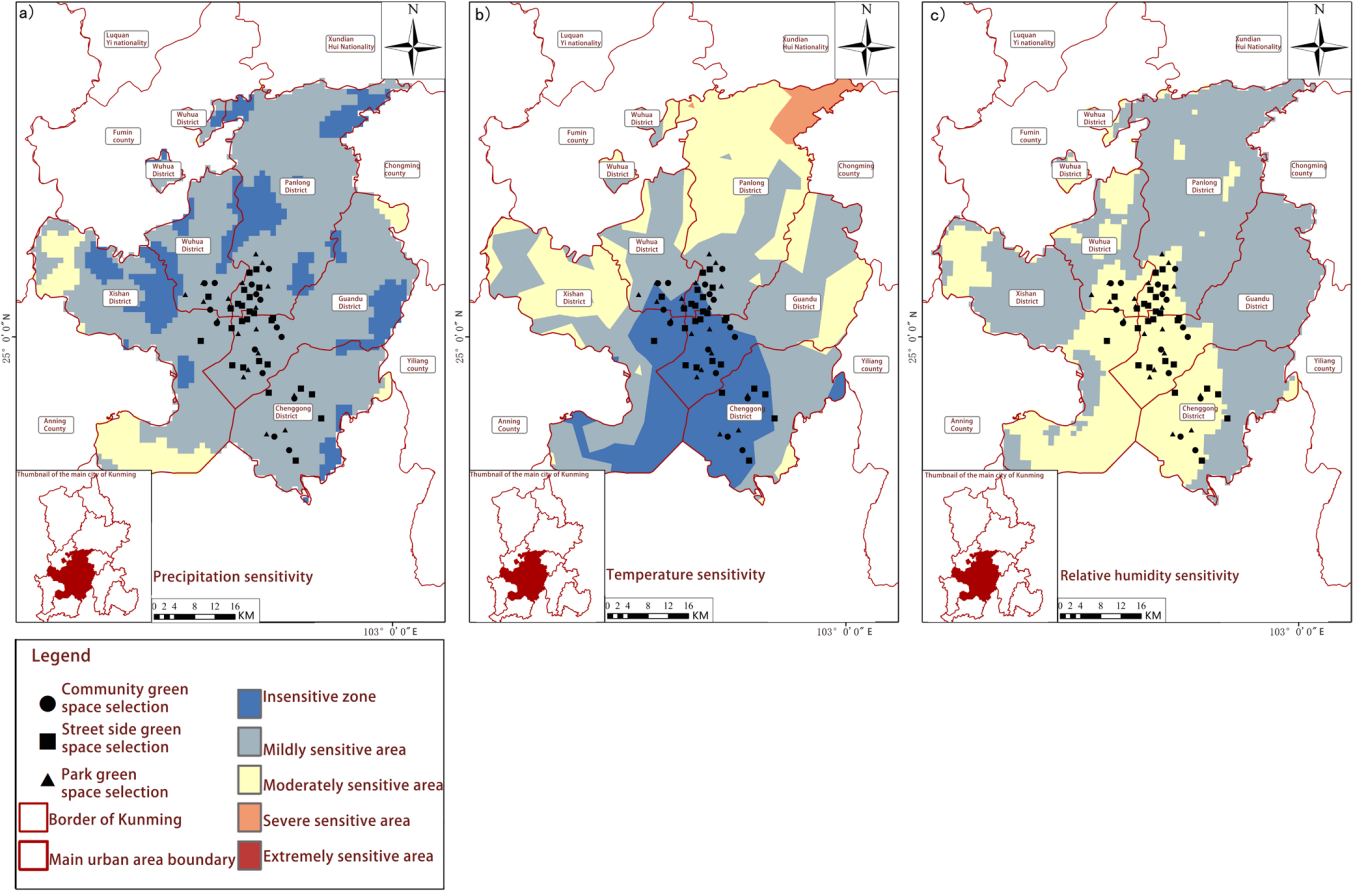
Spatial distribution of climate sensitivity. (a) Precipitation sensitivity; (b) Temperature sensitivity; (c) Relative humidity sensitivity.

**FIGURE 8:**
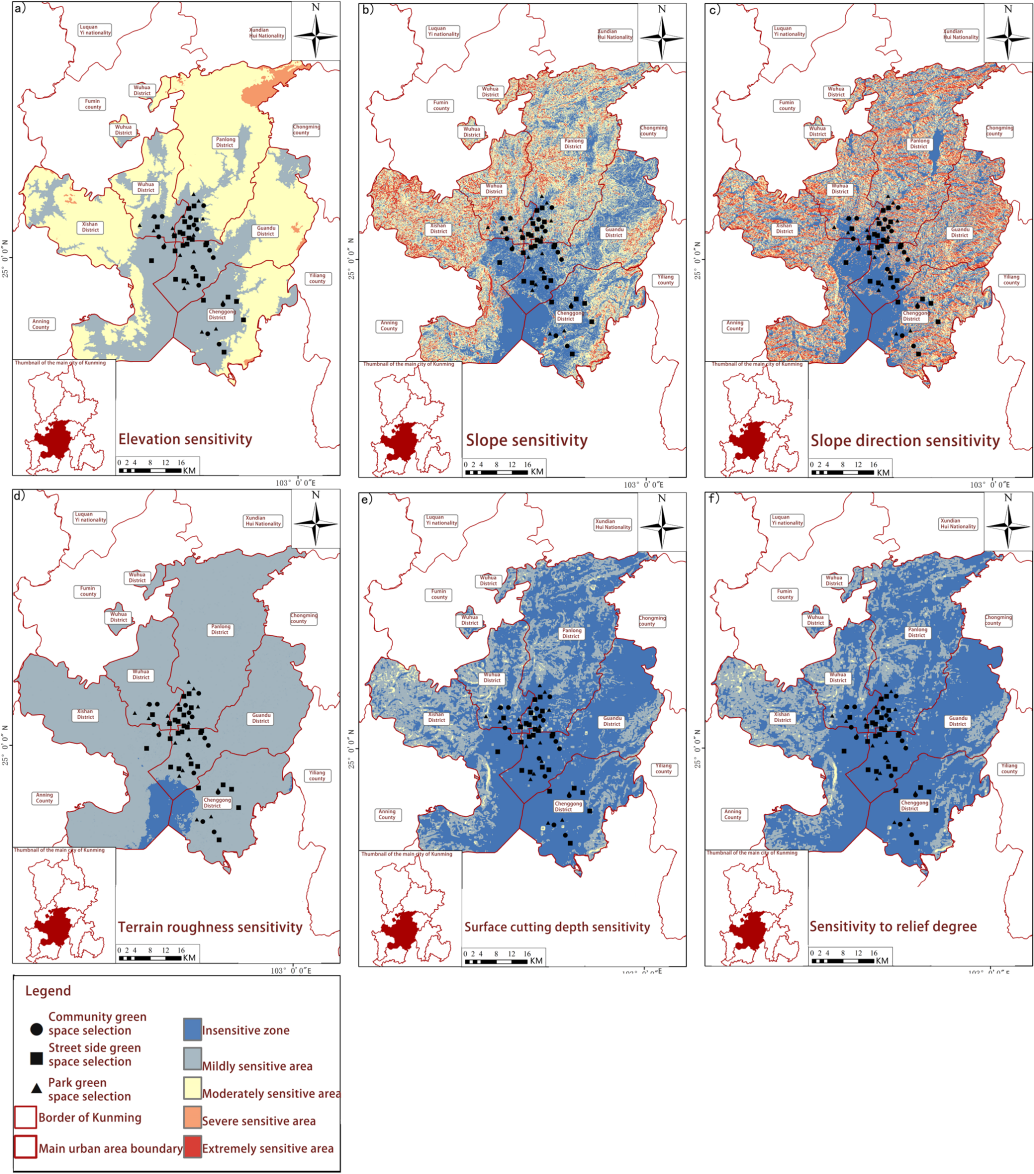
Spatial distribution of geological sensitivity. (a) Elevation sensitivity; (b) Slope sensitivity; (c) Slope direction sensitivity; (d) Terrain roughness sensitivity; (e) Surface cutting depth sensitivity; (f) Sensitivity to relief degree.

**FIGURE 9:**
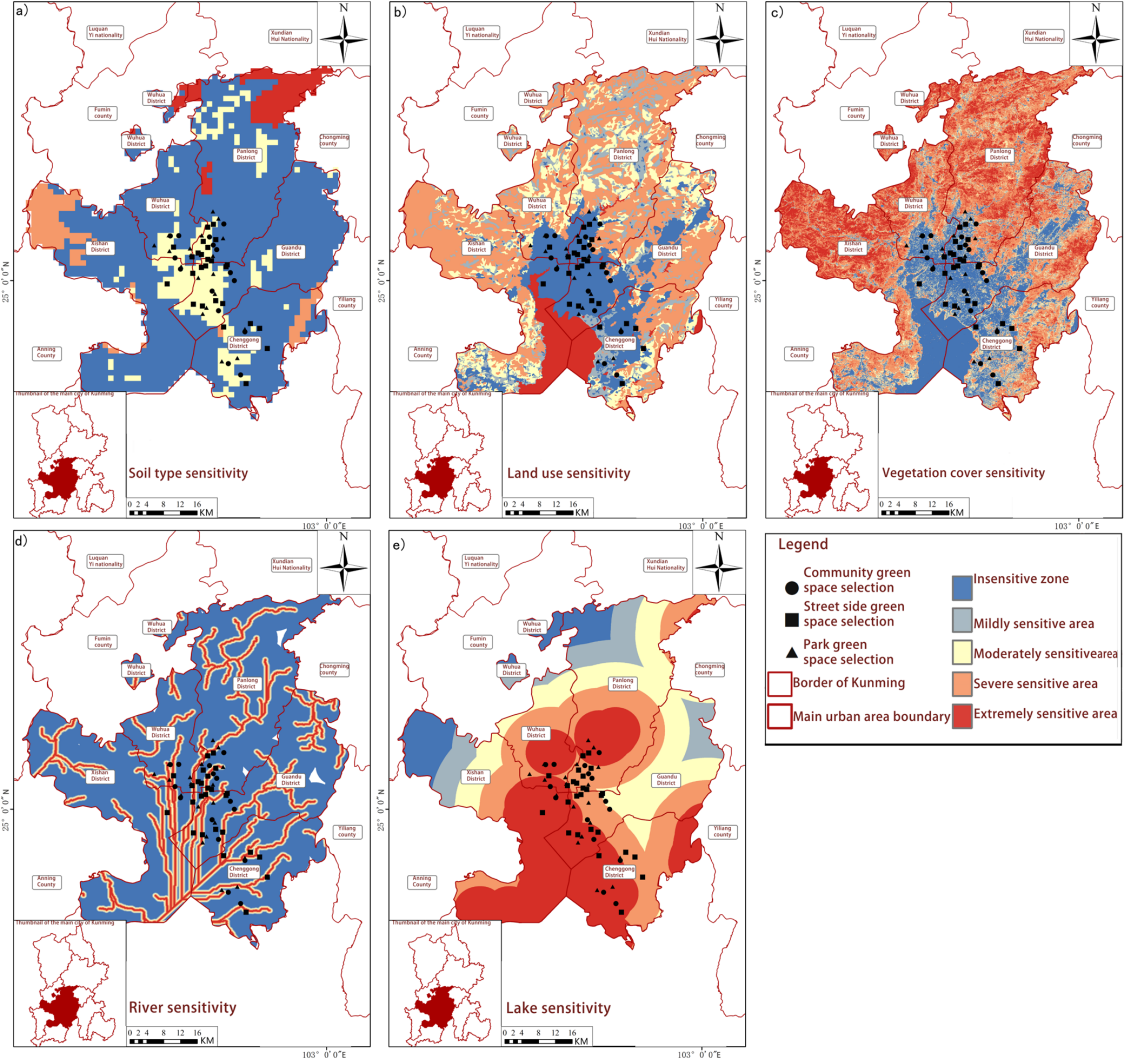
Spatial distribution of natural resource sensitivity. (a) Soil type sensitivity; (b) Land use sensitivity; (c) Vegetation cover sensitivity; (d) River sensitivity; (e) Lake sensitivity.

**FIGURE 10:**
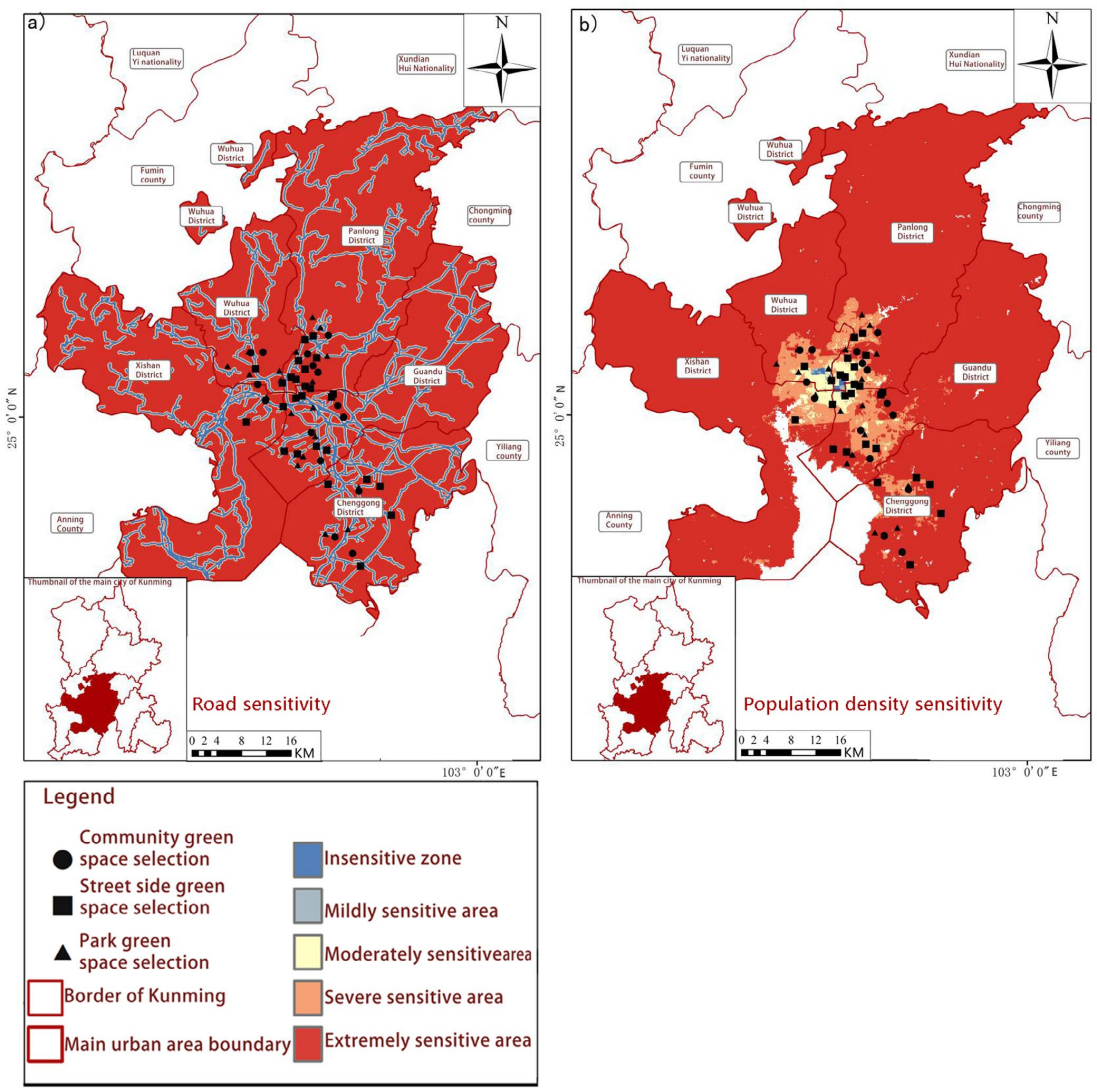
Spatial distribution of human interference sensitivity. (a) Road sensitivity; (b) Population density sensitivity.

Ecological Sensitivity Patterns of Butterfly Habitats in Kunming. The study systematically investigated the ecological sensitivity patterns of butterfly habitats across three distinct urban green space typologies in Kunming City, integrating multidimensional environmental factors. Point‐based data from five core urban districts combined with ArcGIS‐based spatial analysis enabled examination of climatic variables (precipitation, temperature, humidity), geological characteristics (elevation, terrain roughness), natural resource availability (soil type, lake distribution) and anthropogenic disturbances (road density, population density) (Figures 7, 8, 9, 10). Weighted overlay analysis generated comprehensive ecological sensitivity scores, revealing significant variations in habitat sensitivity among different types of green spaces.

Park ecosystems demonstrated heightened sensitivity to climatic variables (precipitation, temperature, humidity) and natural resource availability. Spatial analysis indicated that precipitation‐ and temperature‐sensitive zones (Figure 7) closely corresponded with park boundaries, suggesting microclimate stability and resource accessibility directly regulated habitat suitability. Streetside greenery exhibited greater vulnerability to anthropogenic disturbances, with areas of high road and population density (Figure 10) forming distinct “disturbance‐sensitive zones” that were prone to habitat fragmentation. Community green spaces exhibited dual sensitivity, responding to both natural resources (elevation, terrain roughness, as shown in Figure 8) and human activities (population density, road density), reflecting their unique transitional role in urban ecosystems. Key ecological drivers, including mean annual precipitation, temperature, humidity, elevation, terrain roughness, soil type, lake distribution, road density and population density, emerged as primary determinants of habitat suitability. These findings aligned with the contribution rates of critical environmental factors derived from MaxEnt modelling, validating the analytical framework. Precipitation‐sensitive areas coincided with zones of high butterfly species richness in parks, highlighting the pivotal role of water availability in shaping habitat quality. Temperature‐sensitive zones overlapping with urban core streetside greenery suggested microclimate alterations (e.g., urban heat island effects) directly impacted butterfly survival.

**FIGURE 11:**
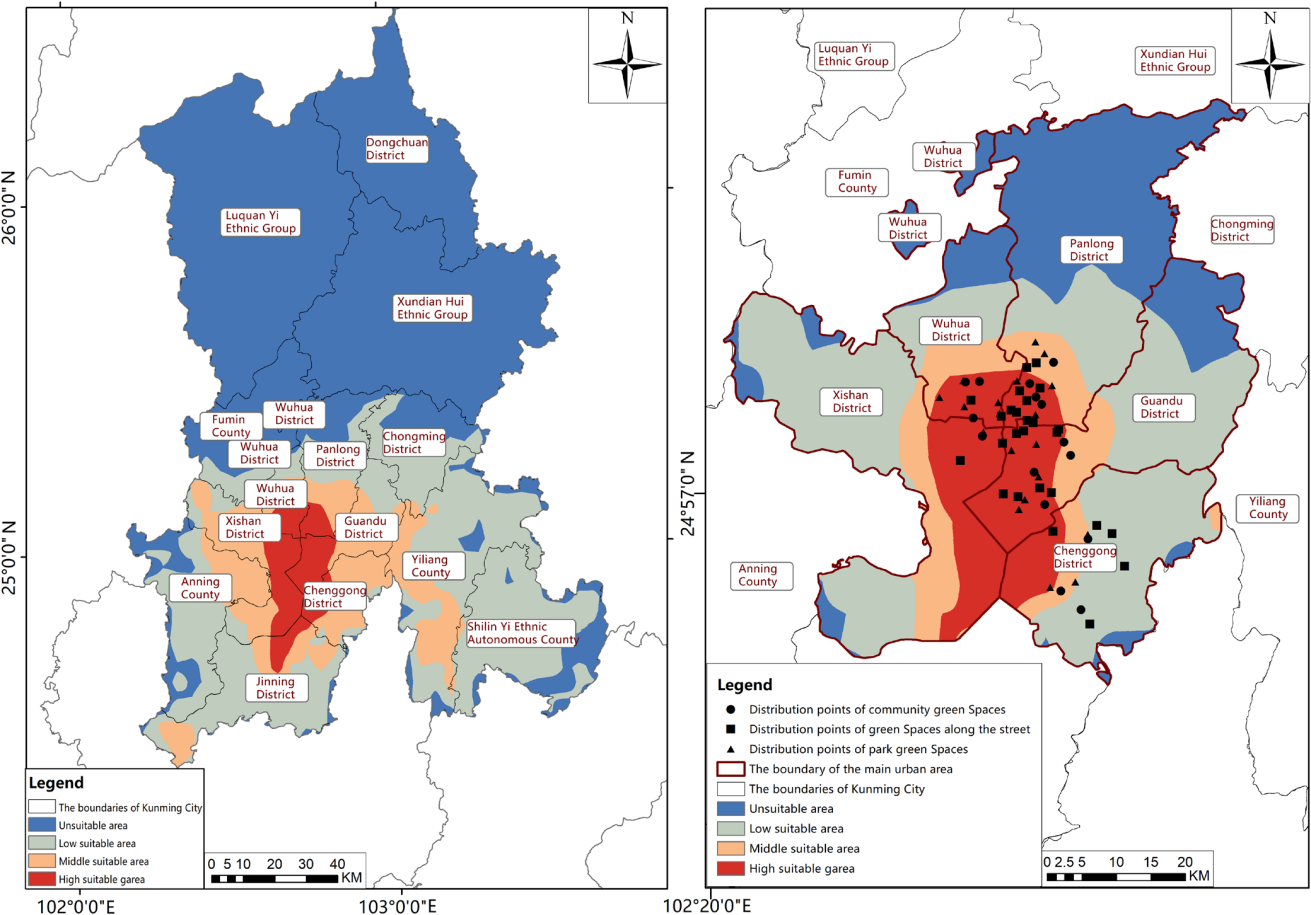
Distribution of butterfly habitat.

**FIGURE 12:**
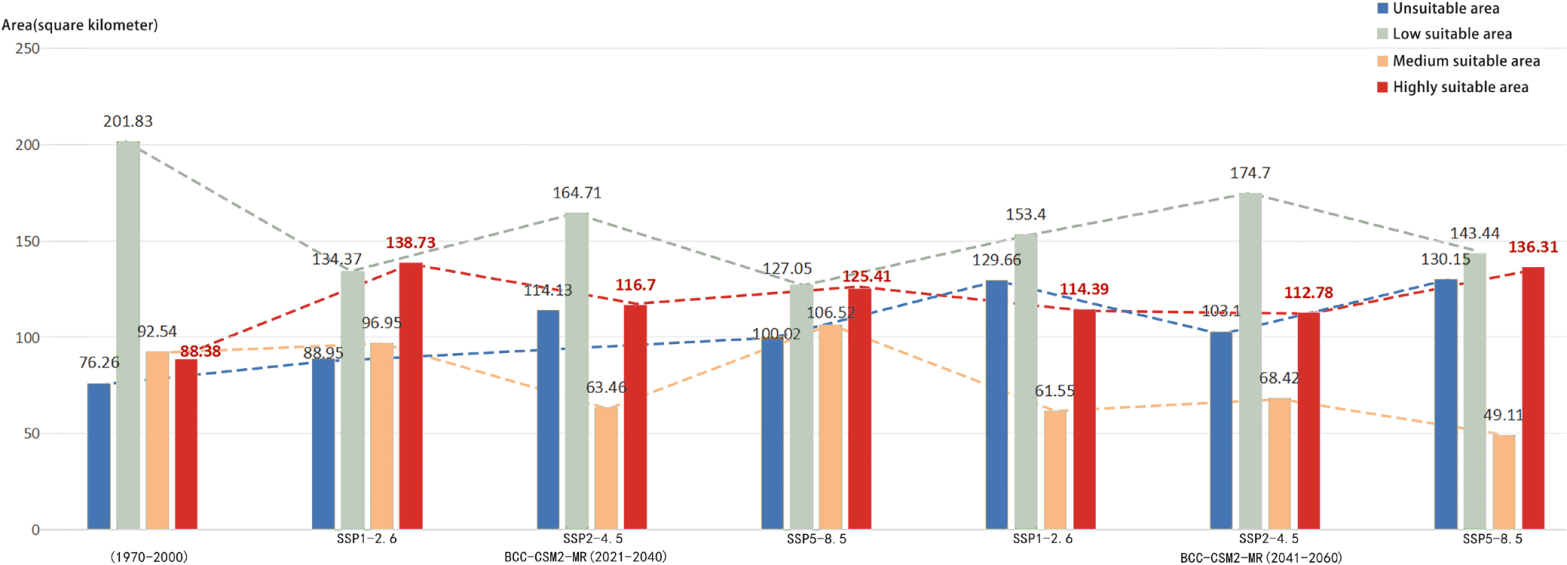
Area of suitable habitat for butterfly species.

**FIGURE 13:**
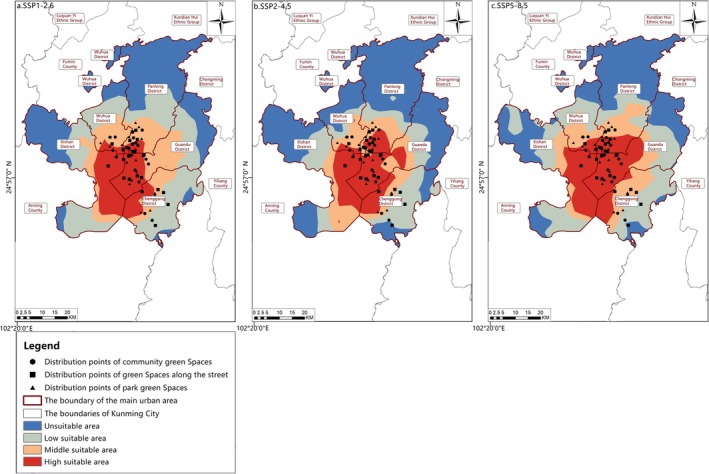
Distribution of suitable areas from 2021 to 2040. (a) SSP1‐2.6; (b) SSP2‐4.5; (c) SSP5‐8.5.

**FIGURE 14:**
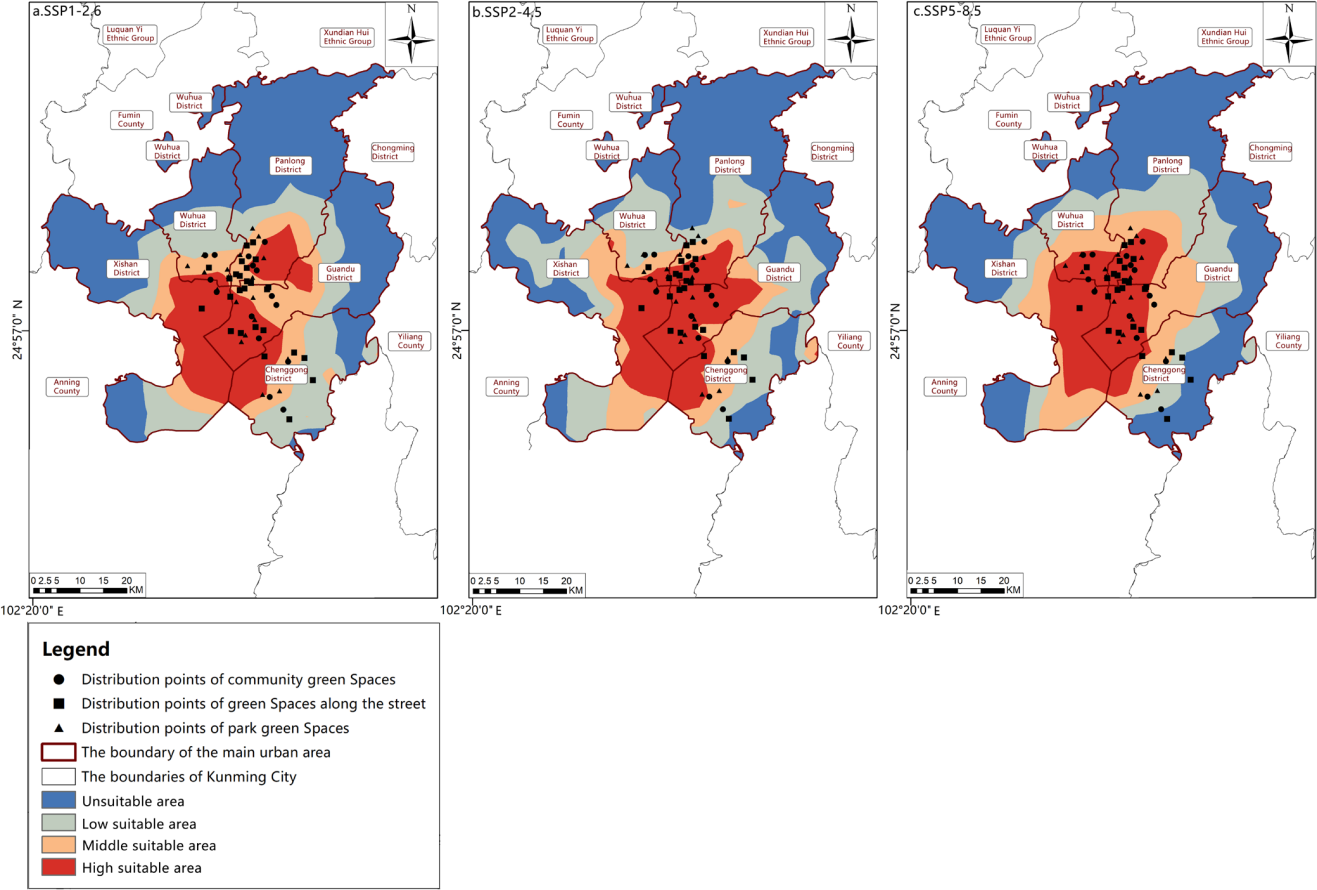
Distribution of suitable areas from 2041 to 2060.(a) SSP1‐2.6; (b) SSP2‐4.5; (c) SSP5‐8.5.

The results emphasise the necessity for tailored conservation strategies, including climate‐adaptive designs for parks to buffer climatic variability, disturbance mitigation measures (e.g., pollution reduction) for streetside greenery, and balanced management approaches for community green spaces. The integration of spatial sensitivity analysis with MaxEnt‐derived factor contributions provides a scientific basis for urban green space planning and biodiversity conservation in Kunming, thereby advancing our understanding of urban ecosystem dynamics. This approach establishes a prototype for multi‐factor sensitivity‐driven methodologies in sustainable urban planning.

### 3.3 Distribution of Suitable Areas

Distribution of Suitable Habitats for Butterfly Species in Kunming (1970–2000). Under the comprehensive climate scenario (1970–2000), the distribution characteristics of suitable habitats for butterfly species in the main urban area of Kunming were distinct (Figure 11). The unsuitable area was predominantly concentrated in the northern part, covering an area of 76.26 km^2^, accounting for approximately 16.5% of the main urban area. The low‐suitable area exhibited a wide distribution across the main urban area, covering an area of 201.83 km^2^, which represented approximately 44% of the total urban area. Moderately suitable areas were primarily located in the south of Wuhua District, the south of Panlong District and the southwest of Guandu District, covering an area of 92.54 km^2^ (20.2% of the main urban area). Highly suitable areas were identified in the southeast of Wuhua District, the southwest of Panlong District, the southwest of Guandu District, the east of Xishan District and the west of Chenggong District, covering an area of 88.38 km^2^ (approximately 19.3% of the main urban area).

Based on the urban green space plots in Kunming during 1970–2000 under this climate scenario (Table 1), eight urban greenbelt plots, namely Luoyang Street Office, Huaxia Tianjing Bay, Langxi Street, Ginkgo Park, Milan Park, Luolong Park, Dayu Park and Gaoxin Neighbourhood, were identified outside the highly suitable areas for butterfly species. Thus, the monitoring of butterfly species in these urban green spaces could be appropriately adjusted to optimise the protection mechanism of urban green spaces in Kunming.

Changes in Suitable Habitat Areas for Butterfly Species in Future Scenarios (2021–2060). Under the BCC‐CSM2‐MR model and different climate scenarios (SSP1‐2.6, SSP2‐4.5, SSP5‐8.5) for the 2030s (2021–2040) and 2050s (2041–2060), significant changes in the area of suitable habitats for butterfly species were observed (Figure 12; Table 7). In the 2030s and 2050s, the unsuitable area showed an upward trend, with the scale varying from 12.69 to 53.89 km^2^. The area of the low‐suitable area decreased, ranging from 27.13 to 74.78 km^2^. Except for the increase in the SSP1‐2.6 and SSP5‐8.5 scenarios in the 2030s, the area of the medium‐suitable area generally showed a downward trend, with a variation range of 24.12–43.43 km^2^. Conversely, the area of the highly suitable area presented an upward trend, ranging from 24.4 to 50.35 km^2^. These changes indicated that, under future climate scenarios, while some types of suitable habitats might shrink, the area of highly suitable habitats for butterfly species in Kunming was expected to expand, which could have potential implications for the conservation and population dynamics of butterfly species in the region.

**TABLE 7:** List of suitable regions for butterfly species.

CMIP6 mode	Time	Climate scenario	Area (Total area 459 km^2^)			
			Uninhabitable area	Low suitability area	Medium suitable area	High suitability area
Synthesis	1970–2000	Synthesis	76.26	201.83	92.54	88.38
BCC‐CSM2‐MR	2021–2040	SSP1‐2.6	88.95	134.37	96.95	138.73
		SSP2‐4.5	114.13	164.71	63.46	116.70
		SSP5‐8.5	100.02	127.05	106.52	125.41
	2041–2060	SSP1‐2.6	129.66	153.40	61.55	114.39
		SSP2‐4.5	103.10	174.70	68.42	112.78
		SSP5‐8.5	130.15	143.44	49.11	136.31

Habitat Suitability Dynamics in the 2030s (2021–2040). Under the BCC‐CSM2‐MR model, although the highly suitable area for butterfly species exhibited an increasing trend in the 2030s, the overall suitable area still showed a decreasing trend (Figure 13; Table 7). Under the SSP1‐2.6 scenario, the suitable area experienced a reduction of 12.69 km^2^. Specifically, the low‐suitable area diminished by 67.46 km^2^, with this decline predominantly concentrated in the southwest of Panlong District and Guandu District. For the SSP2‐4.5 scenario, the suitable area decreased by 37.87 km^2^. Here, the medium‐suitable area decreased by 29.08 km^2^, primarily located in Wuhua District and the southern part of Panlong District. In comparison, the low‐suitable area decreased by 37.12 km^2^, primarily in the western region of Xishan District. Under the SSP5‐8.5 scenario, the suitable area shrank by 23.76 km^2^, with the low‐suitable area decreasing by 74.78 km^2^, concentrated in the eastern part of Chenggong District. By cross‐referencing with urban green space data (Table 1), six types of urban green spaces—namely, Luoyang Street Office, Huaxia Tianjing Bay, Langxi Street, Ginkgo Park, Dayu Park and Gaoxin Neighbourhood—were identified as not falling within the highly suitable areas for butterfly species in the 2030s. Thus, the intensity of their monitoring and protection efforts could be adjusted downward appropriately, streamlining resource allocation for urban green space conservation.

Habitat Suitability Trends in the 2050s (2041–2060). In the 2050s, the BCC‐CSM2‐MR model indicated that, despite the expansion of highly suitable areas, the overall suitable area for butterflies continued to decline (Figure 14). Under the SSP1‐2.6 scenario, the suitable area decreased by 53.4 km^2^. The medium‐suitable area decreased by 30.99 km^2^, concentrated in the southern part of Panlong District and the southwest of Guandu District, while the low‐suitable area decreased by 48.43 km^2^, mainly in the northern regions of Wuhua District and Panlong District. For the SSP2‐4.5 scenario, the suitable area dropped by 26.84 km^2^, with the medium‐suitable area decreasing by 24.12 km^2^ (focused on Wuhua District and the south of Panlong District) and the low‐suitable area reduced by 27.13 km^2^ (concentrated in the northern part of Wuhua District and the northwest of Xishan District). Under the SSP5‐8.5 scenario, the suitable area declined by 53.89 km^2^, with the medium‐suitable area decreasing by 43.43 km^2^ (in Wuhua District and the south of Panlong District) and the low‐suitable area decreasing by 58.39 km^2^ (in the northwest of Xishan District and the northeast of Guandu District). From the urban green space dataset (Table 1), four types of urban green spaces, Luoyang Street Office, Huaxia Tianjing Bay, Langxi Street and Ginkgo Park, were found to lie outside the highly suitable areas for butterfly species in the 2050s. Hence, a targeted reduction in their monitoring and protection efforts was feasible, optimising the efficiency of urban green space conservation measures.

Comprehensive Analysis of Habitat Suitability Changes. Across different climate scenarios simulated by the BCC‐CSM2‐MR model, the highly suitable area for butterflies generally showed an upward trend. In contrast, the overall suitable area exhibited a decreasing trend, with the unsuitable area undergoing more significant fluctuations. The highly suitable area peaked at 138.73 km^2^ under the SSP1‐2.6 scenario in the 2030s. The medium‐suitable area reached a minimum of 61.55 km^2^ under the SSP1‐2.6 scenario in the 2050s. The low‐suitable area reached a minimum of 127.05 km^2^ under the SSP5‐8.5 scenario in the 2030s, and the unsuitable area peaked at 130.15 km^2^ under the same scenario in the 2050s. Overall, based on the spatial distribution of butterfly suitable habitats, the monitoring and protection efforts for four urban green space types (Luoyang Street Office, Huaxia Tianjing Bay, Langxi Street and Ginkgo Park) could be strategically reduced. This adjustment would enhance the cost‐effectiveness of urban green space protection, aligning conservation actions with the dynamic changes in butterfly habitat suitability over time.

## 4 Discussion

### 4.1 Analysis of Butterfly Habitat Suitability Pattern and Its Changing “Paradox”

Using the MaxEnt model, this study assessed the suitability of butterfly habitats in Kunming, revealing marked spatial heterogeneity. Currently, highly suitable areas cluster in the southwest main urban area, including Xishan District, west of Dianchi Lake and Chenggong District, as well as natural mountainous regions such as Xishan Forest Park. These areas, characterised by complex terrain, dense green patches, good connectivity, rich plant diversity and moderate microclimates, provide comprehensive ecological shelter. In contrast, central and northern parts (e.g., Wuhua, Panlong Districts) show low suitability due to fragmented patches, scarce resources and intensified heat (Sun et al. 2012; Tian and Li 2024). Notably, future simulations (2021–2040; 2041–2060) revealed an ecological “paradox”: while total suitable areas declined, highly suitable regions expanded. This stemmed from interactions between urban microclimates and climate change. Marginal areas (e.g., moderately suitable zones) degraded rapidly under climate stress and increased disturbance, whereas ecologically core, high suitability areas maintained or enhanced their carrying capacity through strong regulation and resilience (Zheng et al. 2016).

Kunming's low‐latitude plateau monsoon climate, characterised by large urban temperature differences, intense sunlight and uneven precipitation, exacerbates this issue. Future climate change may prolong droughts and intensify precipitation, exacerbating water deficits and heat stress in moderately suitable areas, leading to habitat contraction. Highly suitable areas, however, benefit from good forest cover, topographic relief and active wind fields, with superior water retention and temperature buffering. Xishan District's mountain woodland landscapes, for example, function as “ecological refuges”, aligning with expanded high suitability zones. The “paradox” highlights the importance of urban green space quality over quantity. Traditional planning focuses on quantity (e.g., green space rate) but neglects quality (Liu et al. 2025).

This study demonstrated that under climate change, green spaces with 3D vegetation, stable hydrothermal conditions, low disturbance and high continuity can sustain butterflies. Thus, preserving the integrity of existing high suitability areas is critical to avoid habitat fragmentation and species isolation (Zhang and Ye 2025; Cao et al. 2025). Current models often use area‐based metrics, overlooking “quality improvement area reduction” effects. This study's “paradox” informs model refinement, emphasising integration of ecological quality, multifactor interactions and microclimate mechanisms for dynamic prediction. Long‐term urban monitoring is necessary to validate model accuracy and ensure its sustainability (Yang et al. 2025). In summary, this study assessed the suitability of Kunming's butterfly habitat, clarified the mechanisms of polarisation and explained climate‐driven “paradoxical” changes. It advances our understanding of urban biodiversity and climate change responses, and supports habitat protection and resilience planning.

### 4.2 Butterfly Habitat Suitability Dynamics Under Climate Change

This study systematically evaluated the temporal dynamics of butterfly habitats in Kunming under future climate change scenarios, utilising three representative pathways (SSP1‐2.6, SSP2‐4.5 and SSP5‐8.5) from the BCC‐CSM2‐MR model, with projections for two future periods (2021–2040 and 2041–2060). Results indicated that suitable butterfly habitats in Kunming would undergo a gradual contraction across all emission scenarios, from low (SSP1‐2.6) to moderate (SSP2‐4.5) and high (SSP5‐8.5). Notably, highly suitable areas exhibited fluctuating growth across the three scenarios during both periods, displaying non‐linear characteristics. This suggested that habitat evolution was not a simple linear process but rather involved distinct “threshold responses” and phase‐specific acceleration.

The period from 2041 to 2060 was projected to represent a critical phase of ecological transition. Under the SSP5‐8.5 high‐emission scenario, this period would witness the most pronounced overall decline in habitat suitability—characterised by a dramatic contraction of moderately and lowly suitable areas alongside a significant expansion of highly suitable zones. This paradoxical pattern might be attributed to the intensification of nonlinear effects within the climate system. Kunming's unique plateau lake‐basin topography, coupled with urbanisation processes, had jointly shaped distinctive local climatic features. Research indicated that the temperature‐moderating effect of Dianchi Lake reduced temperature fluctuations by 2°C–3°C in surrounding areas, while topographic barriers created an east–west precipitation gradient of 200–300 mm. Concurrently, urban heat island effects elevated annual mean temperatures in urban cores by 1.2°C–1.8°C compared to suburban areas, forming specialised “urban thermal oasis” habitats. Climate model projections suggested accelerated increases in global greenhouse gas concentrations, persistent temperature rises and more frequent extreme weather events during this period, which would disrupt the hydrothermal equilibrium of Kunming's plateau monsoon climate and trigger rapid ecosystem destabilisation (Zhu et al. 2015; Li et al. 2017).

Climate modelling analyses identified regional monsoon system variability as the primary driver. The weakening Indian monsoon reduced moisture transport during wet seasons by 10%–15%, while the northward shift of the subtropical high prolonged dry seasons by 12–18 days. These changes exerted differential impacts across ecosystems: water level fluctuations in Dianchi wetlands affected 60% of waterbird habitats; urban thermal stress decreased butterfly flower visitation frequency by 40%–50%; and altered precipitation patterns reduced understory vegetation regeneration rates in Xishan forests by 15%–20%. Particularly under the combined pressures of rapid urbanisation and heat island intensification, increased drought frequency, enhanced surface impermeability and aggravated physiological stress in vegetation (Lin, Ye, et al. 2025; Hrabovský et al. 2025) would directly constrain the survival space and phenological windows of sensitive species, such as butterflies. Spatial analyses revealed that the most substantial declines in habitat suitability were concentrated in urban fringe development zones and high‐density built‐up areas, including eastern Chenggong, northern Panlong and the vicinity of Changshui Airport. These regions, characterised by fragile ecological baselines, intense anthropogenic disturbance, poor green space connectivity and prioritised planned development, were highly susceptible to becoming “ecological traps” for butterfly populations (Ma et al. 2025). In contrast, the Xishan area, located west of Dianchi Lake and encompassing high‐altitude woodlands and water source protection zones, maintained high suitability across all future scenarios. Its dense natural vegetation, restricted development and complex topography created an “ecosystem refuge” effect, providing critical habitat substrates for sustaining butterfly populations (Tang and Yin 2025; Pu et al. 2012).

Our temporal analysis further revealed that urban habitat changes were not continuous or uniform but exhibited distinct “ecological abruptness”. This posed new challenges for urban ecological management, emphasising the need to move beyond reliance on annual average trend projections. Instead, stage‐specific early warning systems, proactive deployment and regionally differentiated response strategies were required to enhance the resilience of urban green space systems against future climate shocks. Thus, future ecological planning must establish a dynamic management framework oriented by temporal sequences and vulnerability, prioritising ecological restoration and adaptive transformation of moderately suitable areas, and explicitly designating the period from 2041 to 2060 as a “priority window” for ecological defence. Measures such as strengthening water conservation, improving green space connectivity and enhancing ecological heat insulation could be implemented to construct urban “ecological resilience buffers” (Lin, Xu, et al. 2025) during this critical period, thereby avoiding irreversible thresholds of habitat degradation.

### 4.3 Environmental Drivers and Butterfly Habitat Response Mechanisms

The study employed the MaxEnt model to simulate the distribution patterns of butterflies in urban green spaces of Kunming, demonstrating high predictive accuracy (AUC = 0.966), which indicates that the selected environmental variables effectively explain the butterfly distribution patterns. Temperature and precipitation emerged as the most influential environmental factors affecting butterfly distribution in Kunming's urban green spaces, with a cumulative contribution rate of 86.5%. The annual temperature range (Bio7) exhibited the highest contribution rate (30.8%) in this study, aligning with global patterns of butterfly sensitivity to temperature fluctuations (Alexander et al. 2013; Zhang et al. 2022). This pronounced effect in Kunming likely stems from the interplay between regional climatic features and species‐specific biological adaptations. As a transitional zone between subtropical and temperate climates, the area experiences marked annual temperature variations (10°C–12°C) that critically influence butterfly life history strategies. Kunming's unique plateau monsoon climate yields pronounced seasonal temperature variations, which directly influence the growth, development and reproductive activities of butterflies. The precipitation of the driest quarter (Bio17) contributed 21.5%, highlighting water availability as another critical limiting factor for butterfly distribution. Under climate change scenarios, increased frequency of extreme high‐temperature events and prolonged drought duration may negatively affect butterfly populations, including accelerated larval development and reduced adult lifespan (Cheng et al. 2025; Wang et al. 2025).

Topographic factors (total contribution rate 6.2%) and anthropogenic disturbance factors (4.8%) showed relatively minor influences. The limited impact of topographic factors may be attributed to the narrow elevation range (1890–2000 m) in the study area. However, local terrain features, such as slope aspect, could still indirectly affect butterfly distribution through microclimate regulation. The lower contribution rate of anthropogenic disturbances suggests that urban butterflies exhibit some degree of adaptability to human activities, presenting opportunities for conserving urban biodiversity (Ding et al. 2025). Notably, while forest cover makes a modest contribution, it remains crucial for maintaining high suitability areas.

Based on the findings, butterfly habitat conservation should focus on the following aspects: primary emphasis should be placed on regulating key climatic factors, particularly through vegetation configuration and water resource management to mitigate extreme temperature and drought impacts; urban green space layout should be optimised to establish ecological corridor networks enhancing habitat connectivity; a long‐term phenological monitoring system should be implemented to prevent plant‐butterfly phenological mismatches caused by climate change; moreover, conservation planning should fully consider small‐scale environmental heterogeneity such as microtopography and microclimate (Zhang et al. 2025). Model predictions require validation through long‐term field observations. Future research could incorporate local‐scale factors, such as host plant distribution and microclimate characteristics, to further improve model accuracy. Subsequent studies should prioritise investigating the dynamic response mechanisms of urban butterfly populations under climate change scenarios to provide more comprehensive scientific basis for adaptive conservation strategies.

### 4.4 Climate‐Adaptive Habitat Conservation Strategies

The study revealed three major challenges facing urban butterfly habitats in Kunming: spatial compression, degradation of quality and climate‐related impacts. Simulation results demonstrated stabilisation trends in high suitability areas, but significant transformation and degradation in medium‐ to low suitability zones. To address this, we propose a three‐tiered protection system: (1) core reserves focusing on strict native vegetation conservation in high suitability areas like Xishan and Liangwang Mountain; (2) medium‐suitability areas implementing habitat quality improvement through enhanced plant diversity and multi‐layered vegetation structures (Wang 2025); and (3) low suitability areas emphasising ecological corridor networks along river greenbelts and linear parks to enhance habitat connectivity (Zhao et al. 2025; Zhang and Zhang 2025).

At the implementation level, we recommend establishing climate‐adaptive ecological zoning with clearly defined functional orientations: core areas prioritising integrity protection, medium‐suitability areas focusing on quality enhancement and low suitability areas strengthening connectivity. New projects should incorporate pre‐construction ecological impact assessments and compensation mechanisms, with green infrastructure integrated into planning standards. Research indicates that even modest (5%–10%) suitability improvements can significantly reduce local extinction risks (Chen and Ma 2010). Ultimately, a multi‐stakeholder collaborative mechanism is crucial: incorporating butterflies into urban ecological indicators at the policy level, advancing population monitoring and response mechanism research through scientific methods, and increasing public awareness through environmental education (Xia et al. 2025). This integrated “core protection–quality improvement–connectivity optimization–public participation” strategy provides both a scientific basis and practical pathways for building climate‐resilient urban ecosystems.

### 4.5 Research Limitations and Future Directions

This study is constrained by its focus on Kunming's urban core, which may limit the generalizability of the results to other subtropical plateau cities. Future research should expand sampling to peri‐urban and rural areas to capture broader landscape dynamics. Additionally, the exclusion of biotic factors (e.g., host plant distribution, predator–prey interactions) represents a gap; integrating these with abiotic variables could refine habitat suitability models (He et al. 2024). Long‐term monitoring of butterfly populations in identified core habitats is crucial for validating model projections and evaluating the effectiveness of conservation measures. Furthermore, exploring the dual role of butterflies—as pollinators in green spaces and potential crop pests in agricultural landscapes—could inform balanced management strategies that bridge ecological protection and agrarian sustainability (Lin et al. 2024).

## 5 Conclusion

This study addressed a critical methodological gap in predicting butterfly habitat suitability under the dual pressures of rapid urbanisation and climate change in subtropical montane cities. By integrating MaxEnt modelling with GIS‐based ecological sensitivity analysis, we systematically deciphered the spatiotemporal dynamics of butterfly habitats in Kunming. Key findings revealed that thermal fluctuations (annual temperature range, Bio7: 30.8% contribution) and precipitation variability (driest quarter precipitation, Bio17: 21.5%) were the dominant drivers of habitat suitability, reflecting the physiological adaptations of butterflies to the hydrothermal regimes of plateau monsoon climates. Spatially, core suitable habitats in southwestern Guandu, northwestern Chenggong and northeastern Xishan districts demonstrated remarkable resilience across both historical (1970–2000) and future (2021–2041, 2041–2060) periods. However, climate projections uncovered a habitat paradox: while highly suitable areas may expand (reaching 138.73 km^2^ under SSP1‐2.6 in the 2030s), total suitable habitats could decline by 53.89 km^2^ under SSP5‐8.5 by the 2050s, highlighting the compounded stress of urban heat islands and extreme climate events on marginal habitats. Methodologically, the coupling of MaxEnt with multidimensional sensitivity analysis (mean AUC = 0.966) overcame limitations of conventional single‐model approaches and achieved 94% field validation accuracy. The model quantified critical ecological thresholds (e.g., annual temperature variation: 19.5°C–21.5°C; dry‐season precipitation: 35–45 mm), providing measurable benchmarks for habitat restoration. Notably, high suitability refugia (e.g., Xishan mountainous woodlands) persisted due to microclimate stabilisation through vegetation cover and topographic modulation, aligning with the global “microrefugia” theory (Hannah et al. 2014; Bergerot et al. 2010). Conversely, moderate‐suitability zones in urban expansion corridors faced elevated degradation risks, demanding prioritised intervention.

Building on these findings, we propose a tiered conservation strategy: (1) strict protection of core zones (e.g., Xishan Forest Park) to preserve native vegetation; (2) habitat quality enhancement in transitional areas via multilayer planting and water conservation measures; and (3) connectivity restoration in low suitability areas (e.g., Luoyang Subdistrict) through riparian greenways and linear parks. This study not only established a replicable framework for assessing subtropical urban habitats but also directly informed Kunming's “Sponge City” initiative and ecological compensation policies. Future research should incorporate biotic interactions (e.g., host plant distributions) and socioeconomic drivers (e.g., land‐use policies) to refine predictive models and advance adaptive management paradigms.

## Author Contributions

**Xiaoli Zhang:** conceptualization (equal), data curation (equal), formal analysis (equal), methodology (equal), software (equal), validation (equal), visualization (equal), writing – original draft (equal), writing – review and editing (equal). **Jiahai Zhao:** formal analysis (equal), investigation (equal). **Kaiyuan Yi:** formal analysis (equal), investigation (equal), supervision (equal). **Di Yuan:** investigation (equal), resources (equal). **Zhe Zhang:** funding acquisition (equal), project administration (equal), supervision (equal).

## Conflicts of Interest

The authors declare no conflicts of interest.

## Supporting information


**Appendices S1–S4:** ece372300‐sup‐0001‐supinfo.zip.

## Data Availability

The authors confirm that the data supporting the findings of this study are available within Supporting Information. All the required data is uploaded as Supporting Information.
