# Correction to “Spatiotemporal Prediction of Ideal Butterfly Habitats in Kun‐Ming's Urban Green Areas: Enabled by Maxent and ArcGIS”

**DOI:** 10.1002/ece3.72580

**Published:** 2025-12-02

Zhang, X., J. Zhao, K. Yi, D. Yuan, and Z. Zhang. 2025. “Spatiotemporal Prediction of Ideal Butterfly Habitats in Kun‐Ming's Urban Green Areas: Enabled by Maxent and ArcGIS.” *Ecology and Evolution* 15, no. 10: e72300. https://doi.org/10.1002/ece3.72300.

The funding information in the published article is incorrect. The correct funding details are provided below:

**Funding:** The study was supported by the National Natural Science Foundation of China (Grant No. 32560393) and Yunnan Fundamental Research Projects (Grant No. 202401BD070001‐118).

Additionally, in Figures 1, 7, 8, 9, 10, 11, 13, and 14, there were inaccuracies in the geographical labels: “Chongming County” was mistakenly used instead of “Songming County,” and “Anning County” was incorrectly used in place of “Anning City.”

The corrected figures are as follows:

**FIGURE 1:**
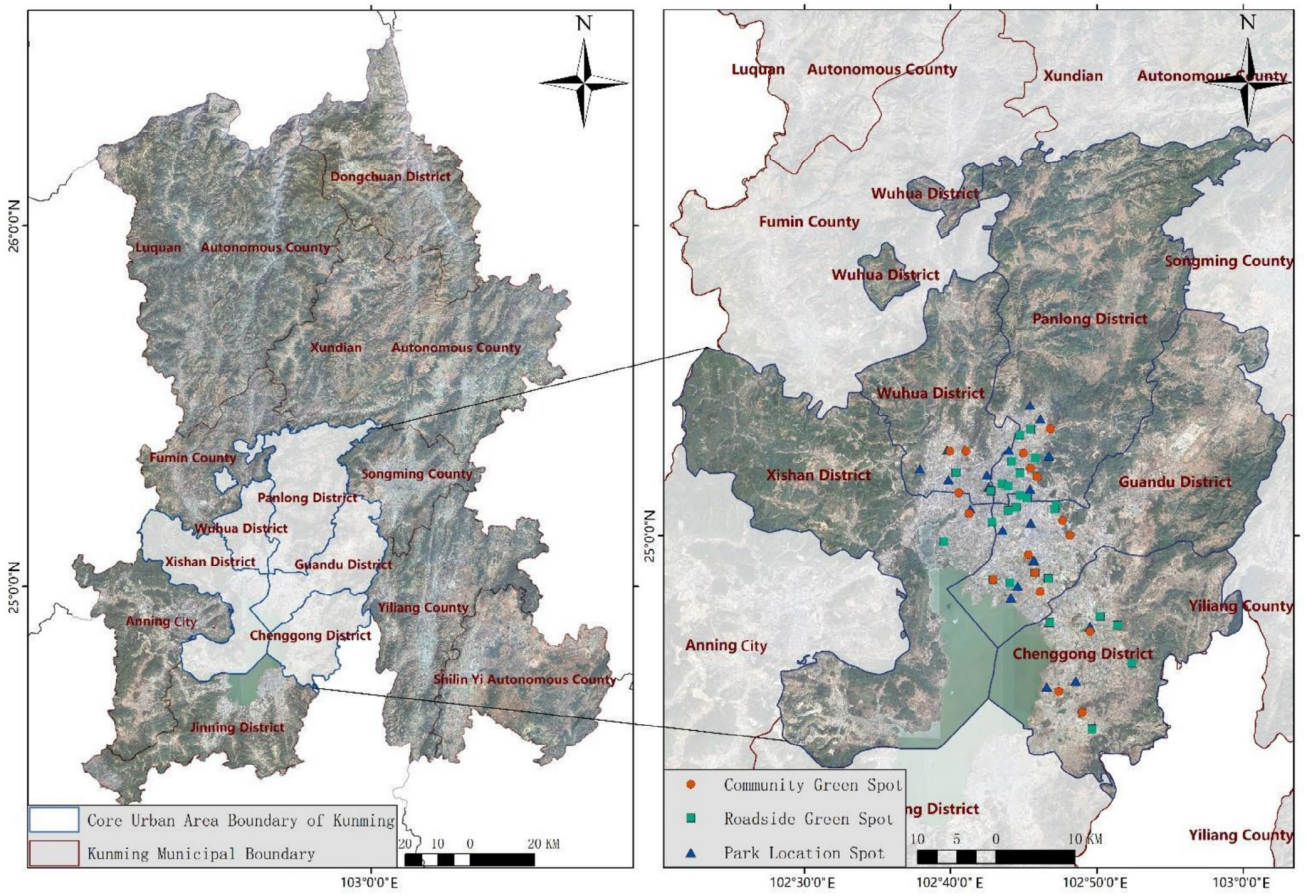
Study area location map. Plan approval number: Reproduction GS (2024) 0650—source osm website (https://www.openstreetmap.org/).

**FIGURE 7:**
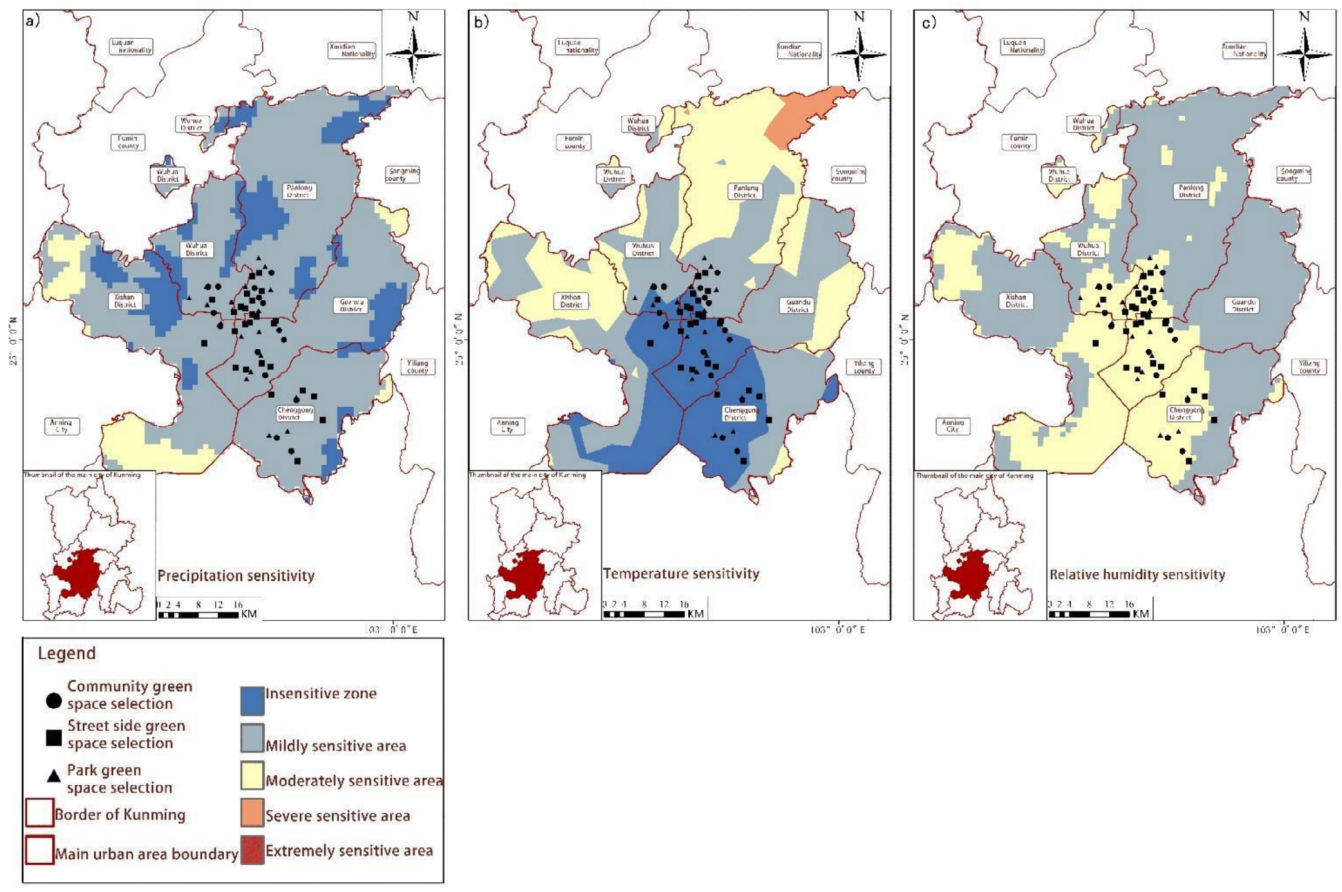
Spatial distribution of climate sensitivity. (a) Precipitation sensitivity, (b) Temperature sensitivity, and (c) Relative humidity sensitivity.

**FIGURE 8:**
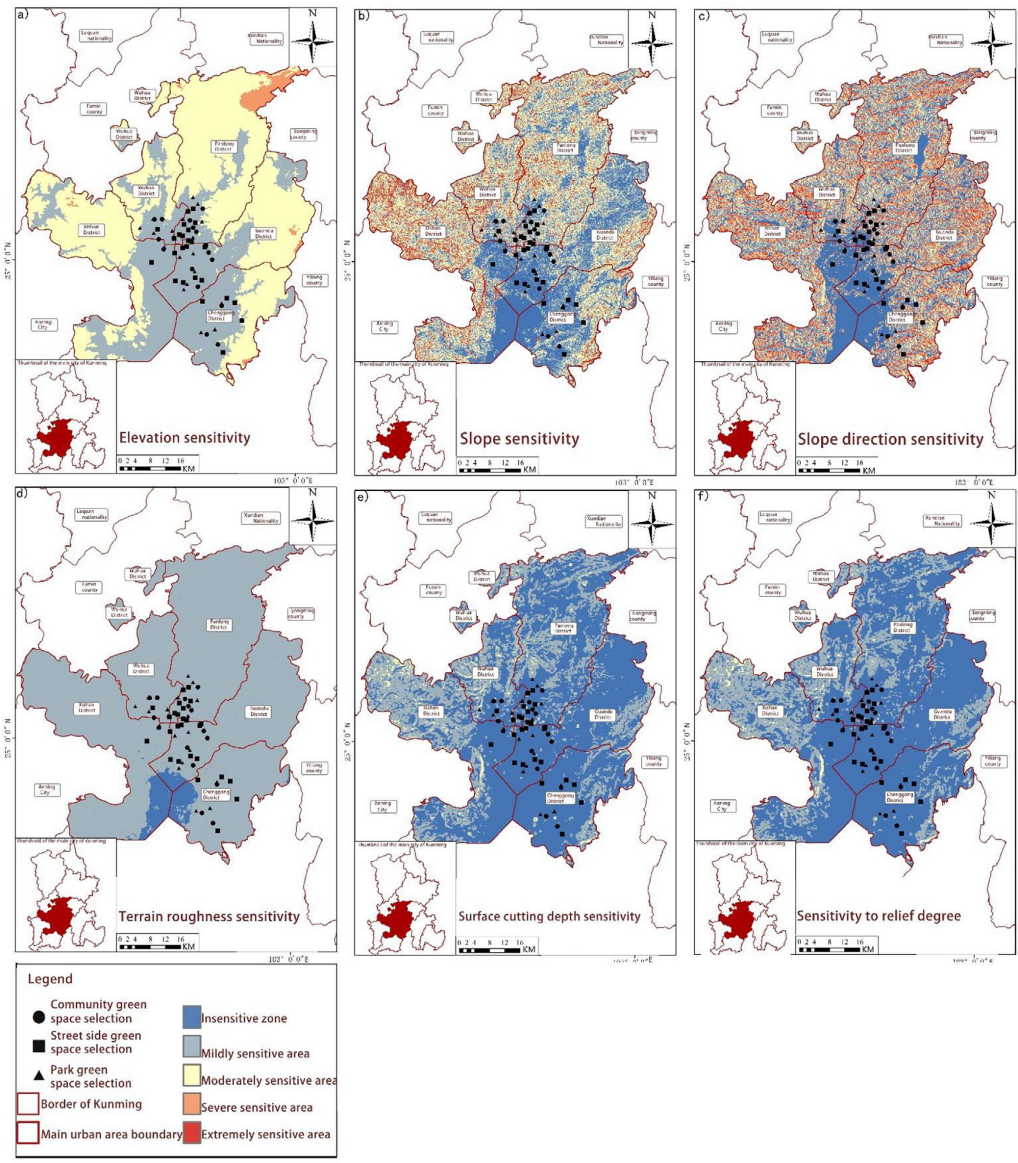
Spatial distribution of geological sensitivity. (a) Elevation sensitivity, (b) Slope sensitivity, (c) Slope direction sensitivity, (d) Terrain roughness sensitivity, (e) Surface cutting depth sensitivity, and (f) Sensitivity to relief degree.

**FIGURE 9:**
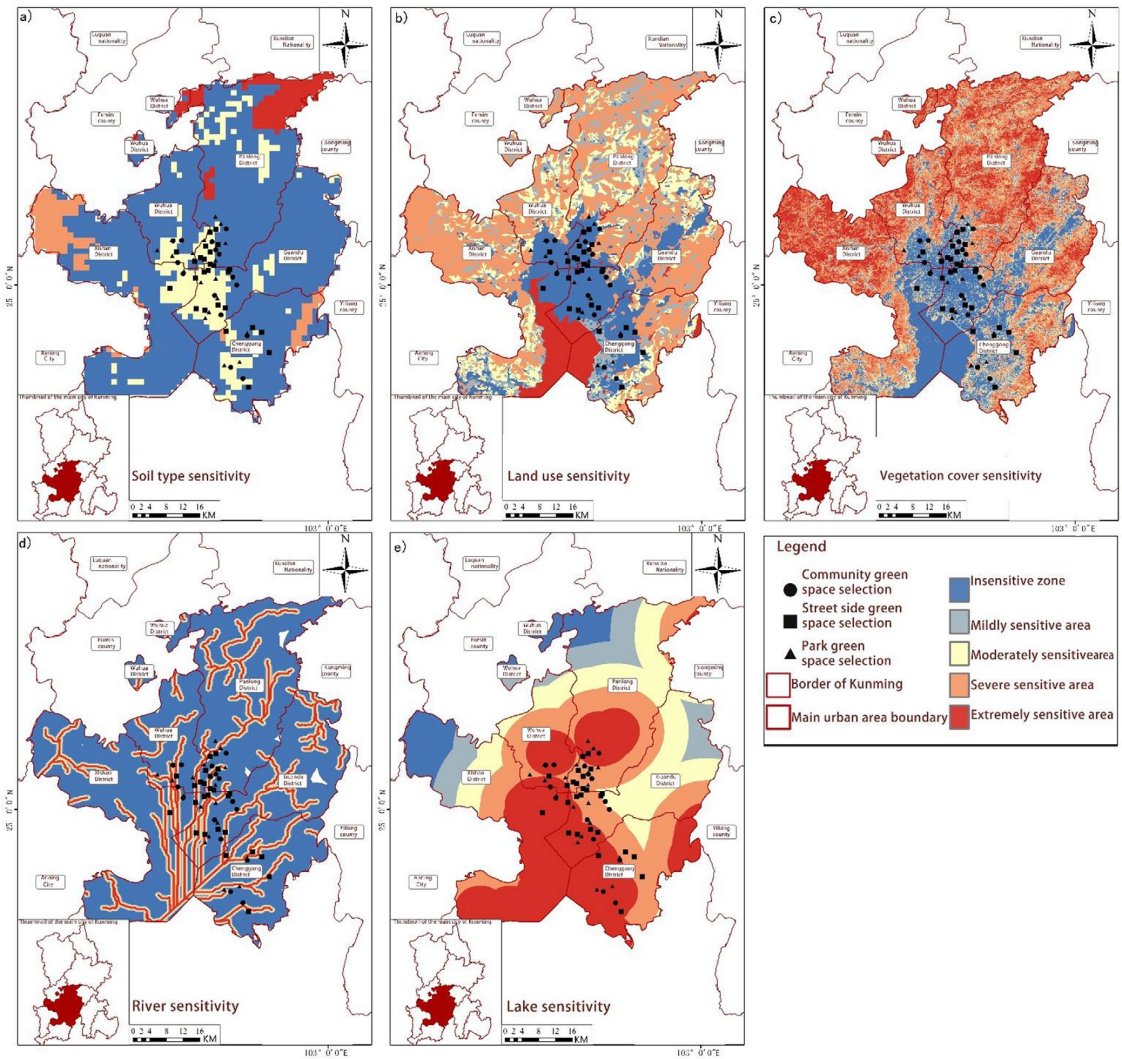
Spatial distribution of natural resource sensitivity. (a) Soil type sensitivity, (b) Land use sensitivity, (c) Vegetation cover sensitivity, (d) River sensitivity, and (e) Lake sensitivity.

**FIGURE 10:**
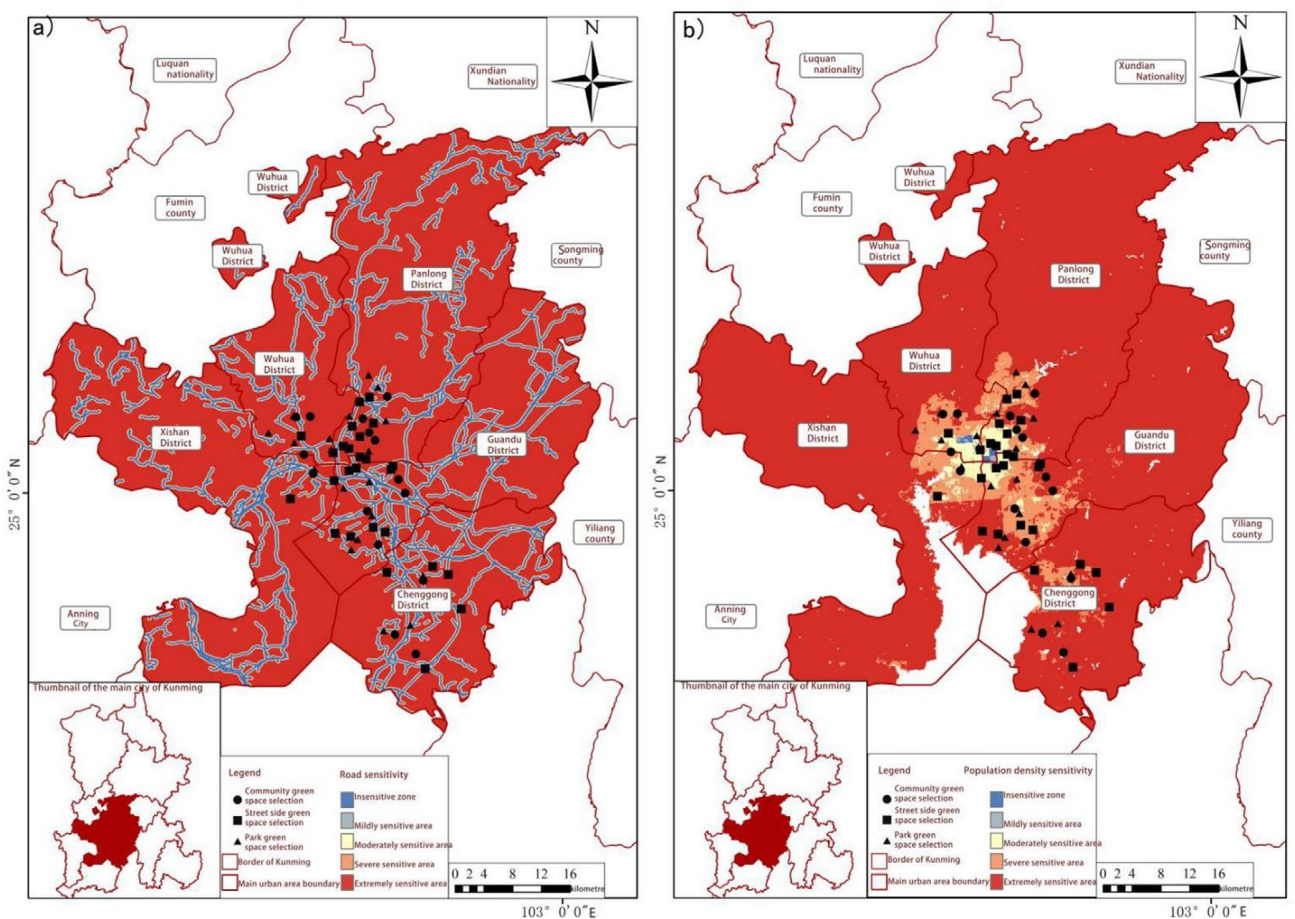
Spatial distribution of human interference sensitivity. (a) Road sensitivity and (b) Population density sensitivity.

**FIGURE 11:**
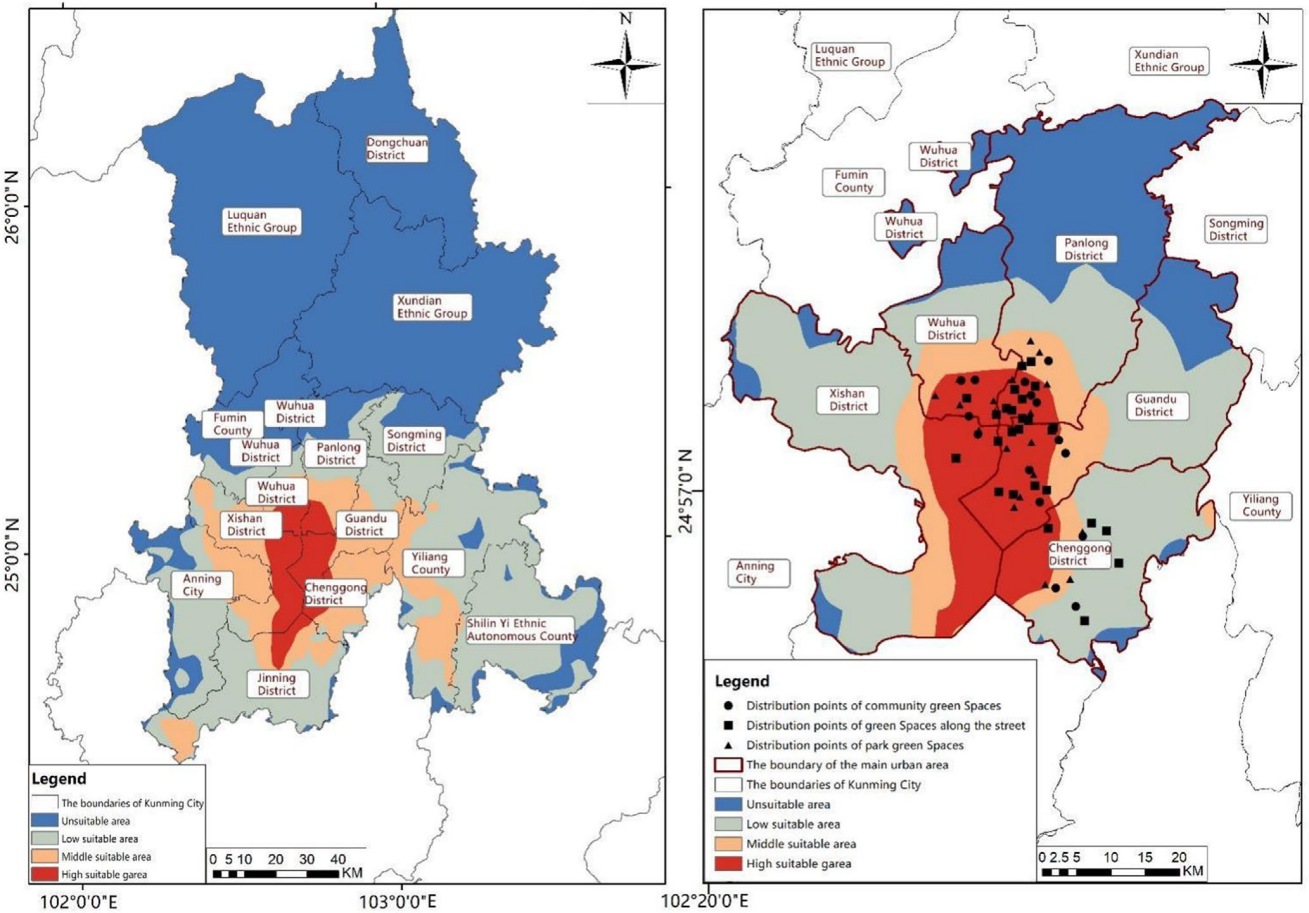
Distribution of butterfly habitat.

**FIGURE 13:**
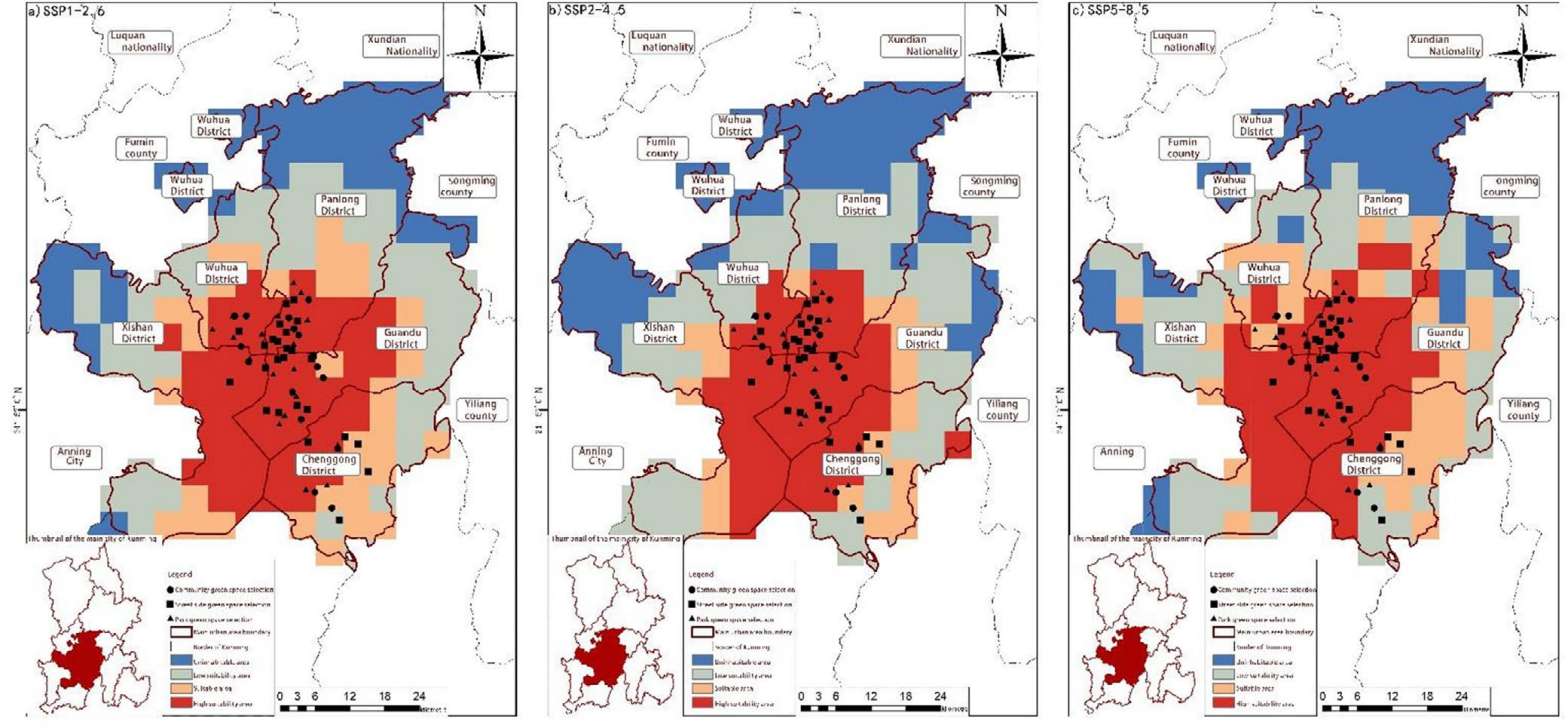
Distribution of suitable areas from 2021 to 2040. (a) SSP1‐2.6, (b) SSP2‐4.5, and (c) SSP5‐8.5.

**FIGURE 14:**
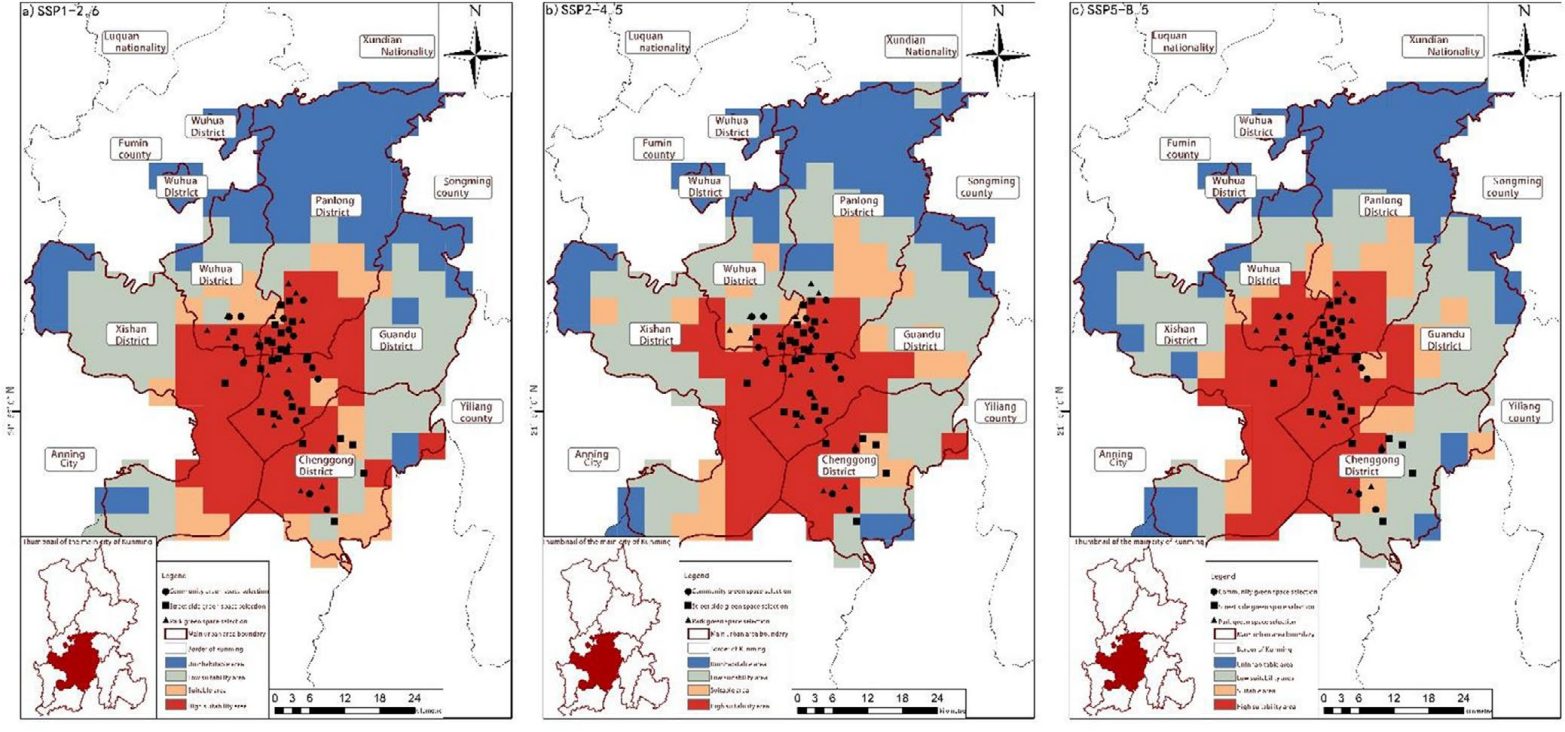
Distribution of suitable areas from 2041 to 2060. (a) SSP1‐2.6, (b) SSP2‐4.5, and (c) SSP5‐8.5.

We apologize for these errors.

